# Recent Advances in Microtubule Targeting Agents for Cancer Therapy

**DOI:** 10.3390/molecules30163314

**Published:** 2025-08-08

**Authors:** Henrique C. Assunção, Patrícia M. A. Silva, Hassan Bousbaa, Honorina Cidade

**Affiliations:** 1 Laboratory of Organic and Pharmaceutical Chemistry, Department of Chemical Sciences, Faculty of Pharmacy, University of Porto, Rua de Jorge de Viterbo Ferreira, 288, 4050-313 Porto, Portugal; henrique.c.a.117@gmail.com; 2 CIIMAR/CIMAR LA-Interdisciplinary Centre of Marine and Environmental Research, University of Porto, Terminal de Cruzeiros do Porto de Leixões, 4450-208 Matosinhos, Portugal; 3 Associate Laboratory i4HB-Institute for Health and Bioeconomy, University Institute of Health Sciences-CESPU, 4585-116 Gandra, Portugal; patricia.silva@cespu.pt; 4 UCIBIO-Applied Molecular Biosciences Unit, Translational Toxicology Research Laboratory, 1H-TOXRUN, IUCS-CESPU, University Institute of Health Sciences, 4585-116 Gandra, Portugal; 5 UNIPRO–Oral Pathology and Rehabilitation Research Unit, IUCS-CESPU, University Institute of Health Sciences, 4585-116 Gandra, Portugal

**Keywords:** cancer, microtubule-targeting agents, natural products, structure-activity relationship studies

## Abstract

Cancer mortality and the development of cancer resistance present significant challenges that must be addressed to ensure global health. Among anticancer agents, microtubule-targeting agents (MTAs) represent a well-recognized therapeutic approach that disrupts microtubule dynamics, thereby inhibiting cell division, and has been widely used to treat several types of cancers. However, even though MTAs are widely regarded as effective, their potential is limited primarily due to cancer resistance and toxicity. Consequently, in the last years, the exploration of new MTAs with the aim of identifying agents with improved cytotoxicity, selectivity, and adequate pharmacokinetic profile, as well as the ability to evade drug resistance mechanisms, has remained a major concern in the development of anticancer treatment. This review highlights the discovery of new MTAs since 2020, with the goal of understanding the advancements made in this field and its future directions. Special attention is given to structure–activity relationship (SAR) studies that could be important for the discovery of more effective MTAs in the future.

## 1. Introduction

To maintain global health, it is imperative to address the significant challenges posed by cancer mortality and the emergence of cancer resistance. Microtubule-targeting agents (MTAs) are a key component of established treatment strategies that have been widely employed to treat various cancer types [1,2,3,4]. Microtubules are components of the intracellular cytoskeleton framework, being essential for many cellular functions in eukaryotic cells, including cell division and mitosis, the development and maintenance of cell shape, intracellular trafficking, and cell signaling [5]. The role of microtubules in regulating these cellular functions has established them as a critical target for anticancer drug discovery. Most MTAs currently in clinical practice or undergoing clinical trials are natural products or derivatives of natural products, including taxanes, vinca alkaloids, colchicine, epothilones, and combretastatins. Nevertheless, toxicity and cancer resistance are the key factors limiting the potential of MTAs, despite their widespread recognition as effective therapeutic agents [6,7,8]. Accordingly, a key focus in the development of anticancer drugs has been the exploration of novel MTAs, including not only those inspired in natural MTAs but also synthetic compounds, with the aim of identifying agents with enhanced cytotoxicity and selectivity, adequate pharmacokinetic profile, and the capacity to elude drug resistance mechanisms. Unlike previous reviews on this topic which focused on few single sites or compound class [7,9,10,11,12], we take a broader approach to new MTAs, including compounds developed for the seven well-established binding sites currently recognized for MTA binding, such as taxane, vinca alkaloids, colchicine, maytansine, laulimalide/peloruside A, pironetin, and gatorbulin binding sites. Additionally, we explore both natural and synthetic MTAs of different chemical class, expanding beyond the traditional emphasis on natural products and their derivatives, in contrast to previous review [13,14,15]. Herein, after a brief description about the microtubule’s structure and dynamics, the well-established binding sites of MTAs, and MTAs resistance, the new MTAs obtained from natural sources or synthesis with antitumor activity discovered since 2020 will be reviewed, highlighting structure–activity relationship (SAR) studies that could be important for the discovery of new MTAs in the future.

## 2. Microtubule’s Structure and Dynamics

**Figure 1:**
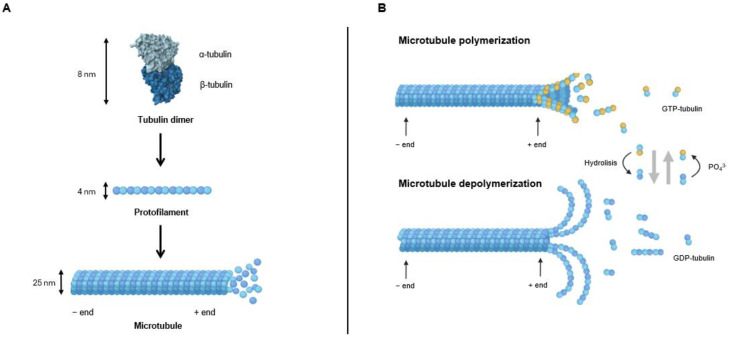
Representation of microtubule structure (**A**) and dynamics (**B**).

Microtubules are integral cytoskeleton components, playing a major role in intracellular trafficking, cell organization, and the formation of mitotic spindle, cilia, and flagella [16,17]. They are composed of α,β-tubulin dimers, which are organized into 13 protofilaments, that connect laterally to form a long, polar, cylindrical structure with a plus and minus end, where fast and slow growth, respectively, occur (Figure 1) [17].

The polymerization and depolymerization of α,β-tubulin dimers is regulated by guanosine triphosphate (GTP) hydrolysis and interactions with microtubule-associated proteins (MAPs) (Figure 1) [17]. Microtubule growth and shrinkage are driven by the incorporation of GTP-bound α,β-tubulin dimers at the plus end. The GTP cap, present at the growing plus end, stabilizes the structure; however, upon GTP hydrolysis to GDP, microtubules become unstable and prone to depolymerization, leading to catastrophe and a rapid transition to shrinkage [18]. Conversely, rescue occurs when microtubules regain a GTP-bound tubulin cap, allowing regrowth. Microtubules exhibit two main types of nonequilibrium dynamics: dynamic instability and treadmilling. Dynamic instability refers to the stochastic switching between growth and shrinkage at individual microtubule ends, with the plus end being more dynamic than the minus end. Treadmilling, in contrast, is characterized by net polymerization at one end while being balanced by net depolymerization at the other, maintaining overall microtubule length despite continuous tubulin turnover. In contrast to the highly dynamic plus end, the minus end is typically anchored to the microtubule-organizing center (MTOC) in human cells, restricting its independent dynamics [18].

## 3. Microtubules as a Prime Target in Cancer Therapeutics

Microtubules have widely been regarded as a primary target of anticancer agents because of their importance in various cellular functions [19]. In fact, MTAs represent an essential part of the chemotherapeutic arsenal. However, these agents have several limitations such as their involvement in multiple cellular functions, unwanted side effects in noncancer cells, and the development of drug resistance [20].

MTAs encompass a broad and diverse collection of antimitotic agents that interfere with microtubule dynamics, which can lead to cell cycle arrest, defects in mitotic spindle assembly, and the activation of apoptotic pathways, ultimately resulting in cell death. The members of this class are usually natural products or derived from natural products, being produced from various sources, including terrestrial and marine organisms, namely, plants, microorganisms, and sponges. MTAs can be classified according to their effects as microtubule stabilizing agents, microtubule destabilizing agents, and microtubule degradation agents [21,22,23].

**Figure 2:**
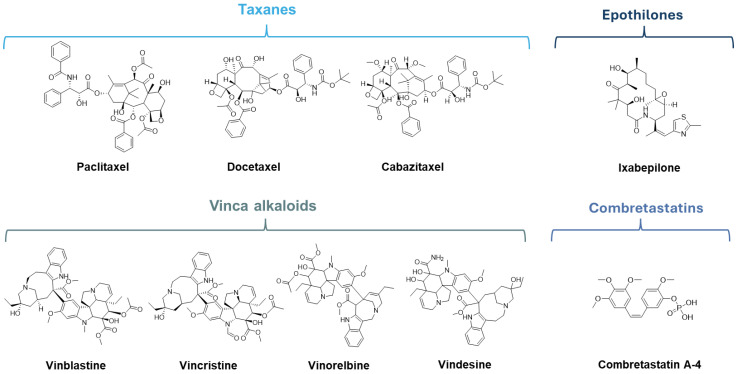
Structure of well-known MTAs.

Microtubule stabilizing agents refer to MTAs that act through the stabilization of microtubules, inhibiting their depolymerization and causing cell death [23]. The best-known representatives of this class, which are presently a part of anticancer therapy, are the taxanes and epothilones [24,25]. Taxanes are polyoxygenated diterpenoids, originally isolated from the yew *Taxus brevifolia,* as well as structure-related semisynthetic analogs [26]. The main representatives of this class present in clinical use are paclitaxel and its semisynthetic analogs docetaxel and cabazitaxel (Figure 2) [27,28]. The epothilones class includes naturally occurring epothilones extracted from the myxobacterium *Sorangium cellulosum* and their derivatives. Epothilone structure is characterized by a 16-membered macrolide ring with a thiazole-containing side chain and an epoxide ring or olefins at C12–C13 [29]. The most noteworthy member of this class, in the field of cancer therapeutics, is ixabepilone (16-aza-epothilone B4, Figure 2), which was approved for the treatment of breast cancer [30].

Microtubule destabilizing agents, despite being very diverse in molecular structure, have in common their effect on microtubules, inhibiting polymerization and leading to their shortening. Vinca alkaloids and colchicine binding site (CBS) inhibitors, such as combretastatins, are important examples of MTAs within this group [31]. Vinca alkaloids, originally isolated from the periwinkle plant *Catharanthus roseus,* are natural or semisynthetic anticancer agents. These compounds comprise a dihydroindole nucleus and an indole nucleus attached through a carbon–carbon bridge [7]. The most prominent members of this group are vinblastine, vincristine, vinorelbine, and vindesine (Figure 2), which were approved for a variety of cancers [32,33,34,35,36,37]. The Combretastatins class is composed of naturally occurring stilbenoid phenols, isolated from the tree *Combretum caffrum*, and their respective analogs. The common combretastatin structure consists of a 3,4,5-trimethoxyphenyl group linked by an olefinic bridge to another phenyl group with frequent substituents on C3 and C4 [38]. The main representatives of this class are combretastatin A4 and its derived prodrug combretastatin A-4 phosphate (Figure 2), which was approved by the FDA as an orphan drug for the treatment of various thyroid cancers and ovarian cancer [15].

Microtubule degradation agents are a novel class of MTAs that exhibit a unique mechanism of action that triggers tubulin degradation [39]. This degradation comes as result of covalent or non-covalent binding of these agents to microtubules, leading to tubulin instability, followed by its depolymerization, disintegration, and aggregation. Tubulin accumulation activates the ubiquitin-proteasome system that will act on tubulin clusters, degrading them. No drugs from this class have yet been approved for cancer treatment, and it is still relatively unexplored but presents a promising field for future research [39].

## 4. Binding Site Landscape of MTAs

The effects of MTAs are mediated through interactions with various binding sites, with seven well-established binding sites currently recognized for MTA binding, namely, the taxane, vinca alkaloid, colchicine, maytansine, laulimalide/peloruside A, pironetin, and the recently discovered gatorbulin binding sites [40,41].

The taxane binding site was first described by Rao et al. (1992) by direct photoaffinity-labeling of tubulin with paclitaxel [42]. It is present in the luminal face of β-tubulin in microtubules, with additional residues occupying α-tubulin [43,44]. Compounds that interact with this active site cause microtubule stabilization [44].

The vinca alkaloid binding site was first described by Gigant et al. (2005) [45] when studying the interaction between vinblastine and tubulin. It is situated at the interface of two tubulin heterodimers arranged head-to-tail, in the vicinity of the exchangeable GTP site [45,46]. Attachment to this site creates a wedge at the junction of α- and β-tubulin and hinders the transition of protofilaments from curved to straight conformation, an essential process for microtubule assembly [46].

The CBS can be found in the α- and β-tubulin interface, adjacent to the GTP N-site of α-tubulin [46,47]. It was originally identified as a result of studying the distinct binding mechanism of the tricyclic alkaloid, colchicine, obtained from the herb *Colchicum autumnale* [48]. Massarotti et al. (2012) divided this binding site into three zones: the first zone in the α,β-tubulin interface, the second zone in an accessory hydrophobic pocket located in the β-tubulin, and the third zone in a deeper binding site in the β-tubulin [49]. Binding to this site impedes microtubule formation by preventing a curved-to-straight conformational change in tubulin [47].

The maytansine binding site was determined by X-Ray crystallography by Prota et al. in 2014 [50]. Maytansine is an ansa macrolide, firstly isolated from the herb *Maytenus ovatus*, that interacts with tubulin inhibiting tubulin assembly into microtubules. The maytansine binding site is in an exposed site of the β-tubulin subunit, adjacent to the vinca alkaloid site, in the interface of two different heterodimers [50,51]. Interaction with the maytansine site leads to microtubule destabilization by two different mechanisms. At low concentrations, it inhibits the connection of new tubulin heterodimers to the plus ends of expanding microtubules. At high concentrations, it captures tubulin subunits into tubulin–drug complexes that cannot agglomerate [52].

The laulimalide/peloruside A binding site was discovered through analysis of the interaction of laulimalide, a macrolide extracted from the marine sponge *Cacospongia mycofijiensis*, with tubulin [53,54]. It is found near the lateral junction between tubulin heterodimers in protofilaments. The binding of laulimalide promotes microtubule polymerization and stabilizes the microtubule structure, leading to the site being classified as a target for microtubule stabilization [54,55].

Pironetin site is located within α-tubulin, where a covalent interaction between the α,β-unsaturated lactone, pironetin, isolated from *Streptomyces* species, and a Cys-316 residue was observed via Michael-type addition reaction [56]. Binding to this site inhibits the connection between tubulin heterodimers, causing a decrease in the addition of new heterodimers to microtubules and leading to destabilization of microtubules [57].

The gatorbulin site was first identified in 2021. This binding site was discovered using X-ray crystallography during the study of the novel cyclodepsipeptide gatorbulin-1, a compound produced by marine cyanobacterium, and its interaction with tubulin [41]. This binding site can be found in the junction between α- and β-tubulin present in the α,β-tubulin heterodimer [41]. The effect of a ligand binding to this site has been described as compound-dependent following observations that indicated that the binding of gatorbulin causes microtubule destabilization, in contrast to cevipabulin, which also targets this binding site but induces tubulin degradation [58].

## 5. MTAs Resistance

One of the growing challenges in using MTAs as anticancer agents is the development of drug resistance. There are three well-described mechanisms responsible for cancer cell resistance to these agents: overexpression of βIII-tubulin, differential expression of β tubulin isotypes, and increased P-glycoprotein (P-gp) activity [59].

The overexpression of βIII-tubulin has been particularly highlighted for its role in MTAs resistance. βIII tubulin, encoded by the *TUBB3* gene, is one of the eight described β-tubulin isotypes, and its upregulation has been reported as a major contributor to MTAs resistance in cancer patients [60,61]. Specifically, clinical observations indicate that high βIII-tubulin expression is associated with resistance to taxane and vinca alkaloid [62]. This resistance arises from the incorporation of the βIII-tubulin isotype into microtubules, which have been observed to exhibit increased dynamic and reduced stability. The exact mechanism causing this change in microtubule dynamics is still unclear, but evidence seems to indicate that the distinct C terminus tail of βIII-tubulin may play a critical role. This enhancement of microtubule dynamics particularly impairs taxane effectiveness, counteracting their ability to suppress microtubule dynamics. Notably, studies have shown that paclitaxel-treated cells containing microtubules enriched with α,βIII-tubulin exhibit significantly lower sensitivity to the drug compared to microtubules composed of various tubulin isotypes [61].

Another factor that might contribute to βIII-tubulin-associated resistance is the less efficient binding of MTAs to this specific β-tubulin isotype. For instance, the weak binding of vincristine and vinorelbine to βIII-tubulin, compared to other isotypes, may account for diminished efficacy in cases of βIII-tubulin overexpression. Additionally, the involvement of βIII-tubulin in oxidative stress has also been observed in ovarian cancer cells, likely due to its effects on oxidative stress response proteins, such us dimethylaniline monooxygenase 4 and glutathione S-transferase 4. However, the biological consequences of this process are still unclear [61,63].

ATP-binding cassette (ABC) membrane efflux pumps have been identified as the main contributors to the development of MTA resistance in cancer cells. Within the ABC superfamily, the primary subfamilies responsible for MTA resistance are the ABCB subfamily, which includes multidrug resistance proteins (MDRs), and the ABCC subfamily, which encompasses multidrug resistance-associated proteins (MRPs) [64,65].

From the MDR class, the transmembrane unidirectional efflux pump P-gp protein, also known as multidrug resistance protein 1 (MDR1), can bind to a broad range of substrates, and its overexpression is associated with the development of resistance to MTAs [66]. Since MTAs, such as taxanes and vinca alkaloids, are substrates of P-gp and permeate the cell membrane through passive diffusion, overexpression of P-gp leads to the active efflux of these drugs, resulting in decreased intracellular drug levels and reduced cytotoxicity. While many MTAs such as taxanes and vinca alkaloids are well-established P-gp substrates and thus vulnerable to efflux-mediated resistance, others avoid this mechanism due to specific physicochemical traits. For example, compounds like colchicine site-binding agents such as combretastatin A-4 and ABT-751 possess lower molecular weights and reduced affinity for P-gp, which allows them to maintain intracellular concentrations even in resistant cells. Similarly, epothilones like ixabepilone, though recognized by P-gp, exhibit less pronounced resistance due to their unique conformational flexibility and partial avoidance of P-gp efflux through weaker binding affinities [67,68]. In addition to MDR1, evidence suggests that MDR3 plays a part in paclitaxel resistance and has been observed to be overexpressed in paclitaxel-treated cells [3]. In the MRP class, MRP1 and, to a lesser extent, MRP2 and MRP7 actively transport and confer resistance to taxanes, while epothilone B is only a substrate of MRP7 [3,69,70]. Differential expression of these transporters contributes to the development of resistance to MTAs in human cancers, but further studies are required to establish precise conclusions.

Resistance to MTAs is also influenced by how specific drugs interact with their binding sites on tubulin. For instance, taxanes bind to the luminal side of β-tubulin and stabilize microtubules by promoting lateral interactions. However, mutations or isotype shifts (e.g., increased βIII-tubulin) can hinder these interactions. In contrast, colchicine- and maytansine-site binders interact with more external or interfacial regions of tubulin, where structural differences among isotypes have a smaller impact, explaining their efficacy in resistant settings. Importantly, SAR studies have shown that small modifications to the binding moieties of CBS agents can dramatically alter their tubulin binding affinity and ability to bypass resistance [49,50].

To overcome resistance, new-generation MTAs have been designed as dual inhibitors. These compounds simultaneously target tubulin and other oncogenic pathways such as kinases (e.g., Aurora inhibitors). For example, tubulin–kinase hybrid molecules have demonstrated enhanced potency and the ability to bypass resistance mechanisms through multi-target disruption. By interfering with both mitotic spindle dynamics and intracellular signaling pathways essential for cancer cell survival, these agents reduce the likelihood of single-pathway resistance emergence. Preclinical studies have shown synergistic cytotoxicity and improved apoptosis induction in resistant cell lines using such strategies [71,72].

## 6. Recent Advances in MTAs

Recently, the field of MTAs has been extensively researched, and consequentially, many new compounds have been developed exhibiting activity against various cancers. This review highlights promising new MTAs developed in the last five years, targeting the well-established binding sites currently recognized for MTA binding. These compounds were categorized according to their binding site and are presented in the chronological order of their report in Section 6.1, Section 6.2, Section 6.3, Section 6.4 and Section 6.5 and in Table S1 (Supplementary Materials).

Most of the MTAs identified in the last five years interact with CBS. However, compounds with affinity to maytansine, taxane, vinca alkaloid, and laulimalide binding sites have also been reported. As far as we know, no new MTAs with proven antitumor activity targeting pironetin and gatorbulin binding sites isolated from natural sources or synthesized in the last five years have been reported.

### 6.1. MTAs Targeting Taxane Binding Site

**Figure 3:**
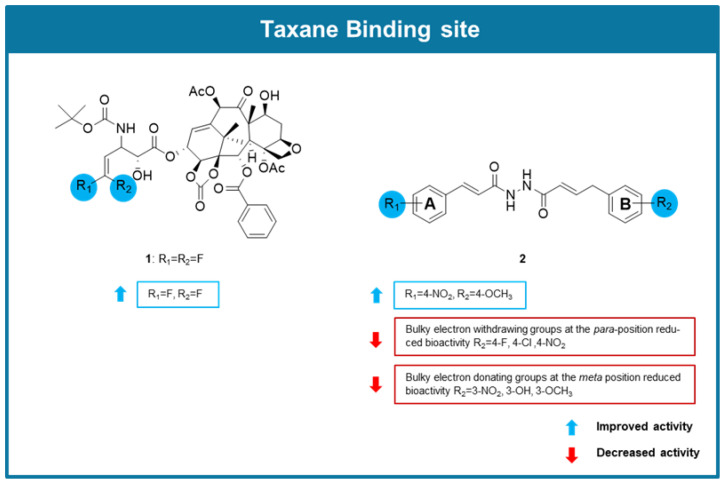
New MTAs with anticancer properties targeting the taxane binding site.

In 2020 Rong et al. developed the novel taxane 3′-difluorovinyl-ortataxel (DFV-OTX, **1**), whose structure is related to the second-generation taxane ortataxel, in which the isobutyl group at the C-3′ position was replaced by a difluorovinyl group (Figure 3) [73]. After successful synthesis, its cytotoxicity was evaluated along with ten novel third-generation taxanes derived from 10-deacetylbaccatin against a paclitaxel-resistant human breast cancer (MCF-7R) cell line. Results showed that **1** had significantly stronger cytotoxic activity in MCF-7R cells than paclitaxel and third-generation taxanes. Further studies were conducted with **1** using a cell-counting kit-8 assay against a panel of breast cancer cell lines, including drug-resistant cancer cell lines. Compound **1** exhibited IC_50_ values lower than paclitaxel in every tested cancer cell line except MCF-7. Through an immunofluorescence assay, **1** was also shown to strongly induce tubulin polymerization in paclitaxel-resistant breast cancer cells, displaying a more potent effect than paclitaxel. Molecular docking studies of DFV-OTX (**1**) in comparison to paclitaxel and ortataxel made it possible to verify that the higher promotion of tubulin polymerization observed for **1** in comparison to paclitaxel is likely due to new H-bonding and van der Waals interactions of the 3′-difluorovinyl group with the taxane binding site. A cell cycle assay further revealed that this compound induced G2/M phase arrest in MCF-7R, MDA-MB-231R, MCF-7, and MDA-MB-231 cells. Additionally, **1** caused apoptosis and endoplasmic reticulum stress at a significantly higher rate than paclitaxel in MCF-7R cells. These results were supported in vivo using PTX-resistant breast cancer tumor xenograft mouse models, as it was observed that the treatment with **1** substantially reduced tumor volume growth of the injected MCF-7R and MDA-MB-231R cancer cell lines [73].

To discover potential anticancer agents based on natural products targeting tubulin with an α,β-unsaturated carbonyl group, *N*-cinnamoyl-N’-(substituted) acryloyl hydrazide derivatives were designed, synthesized, and screened for anticancer activity by Zhou et al. (2022) [74]. Antiproliferative activity evaluation was carried out on adenocarcinomic human alveolar basal epithelial (A549), human prostate cancer (PC-3), and human hepatoma (HepG2) cancer cell lines. From the 26 derivatives tested, compound **2** (Figure 3) showed the most promising results, exhibiting IC_50_ values between 3.36 and 5.99 µM, displaying low cytotoxicity towards normal NRK-52E cells. Analysis of compound **2**′s structure in comparison to other synthesized derivatives indicated that the position of the substituents on the aromatic rings interferes with the activity, being observed that the presence of a *para*-nitrophenyl group on the A aromatic ring, and a bulky OCH_3_ group at the *para* position of the B ring was associated with an enhancement of the activity (Figure 3). An immunofluorescence assay performed on HepG2 cells treated with **2** showed its capacity to induce microtubular aggregation and wrinkling of the cell nucleus. Tubulin polymerization assay and transmission electron microscopy revealed that **2** stabilized tubulin assembly, promoted protofilament assembly, and promoted microtubular aggregation. Molecular docking studies suggested that compound **2** interacted with the taxane binding site. Additionally, a flow cytometry analysis revealed that HepG2 cells treated with **2** showed an increase in G2/M phase arrested cells. Moreover, compound **2** caused A549 cell migration rates to decline and demonstrated a strong apoptotic effect in HepG2 cells [74].

### 6.2. MTAs Targeting Vinca Alkaloids Binding Site

**Figure 4:**
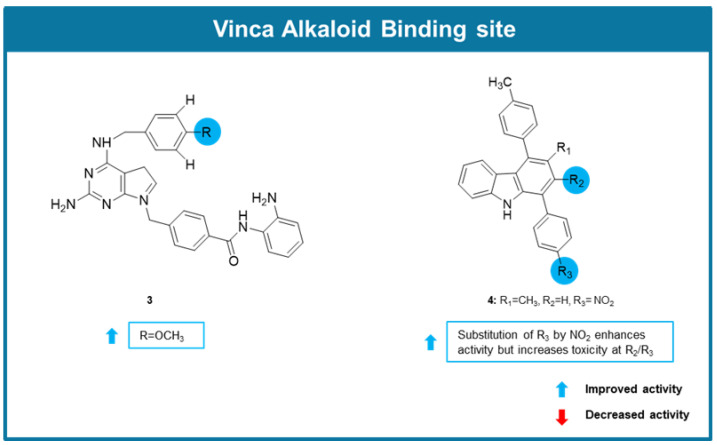
New MTAs with anticancer properties targeting the vinca alkaloids binding site.

In 2021, Singh et al. identified compound **3** (Figure 4) as a dual histone deacetylase (HDAC1)/tubulin inhibitor [75]. A significant cell growth inhibitory effect against a panel of human cancer cell lines was observed for cells treated with compound **3** (IC_50_ values between 0.05 and 0.47 µM). In addition to this, **3** was shown to induce cell cycle arrest apoptosis, being this effect associated with an increase of caspase-3 and -9 and a reduced expression of anti-apoptotic proteins MCL-1 and Bcl-x. The effect on microtubule assembly was assessed by an in vitro tubulin polymerization assay, in which it was confirmed that **3** significantly reduced the efficiency of tubulin polymerization, with similar potency to vinorelbine. Noteworthy, the antiproliferative activity of **3** was maintained in MDR MES-SA/Dx5 cancer cells. Molecular modelling studies allowed to predict the interaction of this compound between β1 and ⍺2 tubulin subunits, like vinolrebin [75].

In the same year, from the search of new potential anticancer agents by Sinicropi et al., the new carbazole **4** (Figure 4) targeting microtubules was identified [76]. This compound displayed promising antiproliferative activity against two breast cancer cell lines, triple-negative MDA-MB-231 and ER (+) MCF-7, showing IC_50_ values lower than 12 µM. SAR studies allowed to conclude about the influence on the presence of a nitro group in the bioactivity (Figure 4). Interestingly, **4** exhibited selectivity for cancer cells, as no cytotoxicity was observed when noncancer cells MCF-10A were exposed to **4**. Immunofluorescence studies using MCF-7 cells allowed to verify that cells exposed to this compound showed microtubule disorganization, like vinblastine, suggesting that **4** could act as a tubulin-polymerization inhibitor. Docking studies predict the interaction of **4** with the interface between subunits α and β in proximity to the vinblastine binding site [76].

### 6.3. MTAs Targeting Colchicine Binding Site

**Figure 5:**
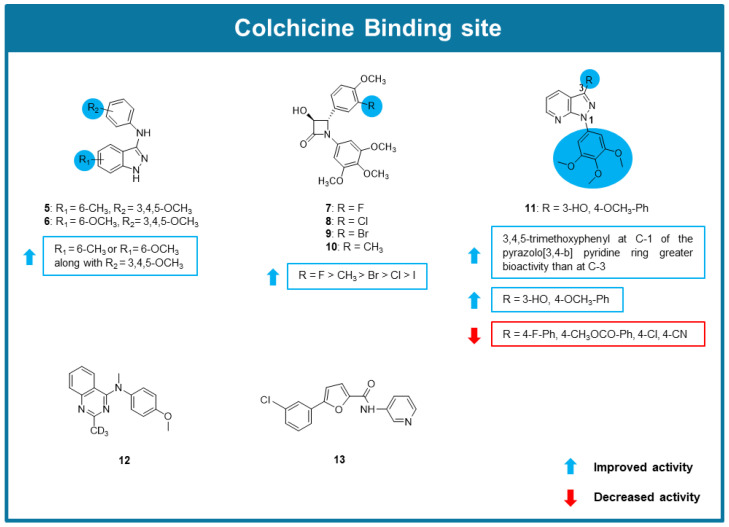
MTAs **5**–**13** with anticancer properties targeting the CBS.

A series of 22 new indazole derivatives were synthesized and screened for their anticancer potential by Cui et al. (2020) [77]. Most indazoles showed promising antiproliferative activity against a panel of cancer cell lines, being compounds **5** and **6** the most potent, with IC_50_ values in the nanomolar range. The SAR studies indicated that a methyl or methoxy substitution and a 3,4,5-trimethoxyphenyl moiety were preferred for the highest antiproliferative activity (Figure 5). The study of the mechanisms underlying their antiproliferative effect demonstrated that **5** and **6** inhibited tubulin polymerization (IC_50_ values of 3.39 and 4.77 µM, respectively) and disrupted cellular microtubule networks, associated with the binding to the colchicine site. These effects were confirmed by *N,N*-ethylenebis(iodoacetamide) (EBI) competition assay, demonstrating the direct interaction of both compounds with the CBS. Moreover, **5** and **6** promoted G2/M cell cycle arrest and induced apoptosis in HCT116 cells. In a HCT116 xenograft mouse model, **5** and **6** reduced tumor growth without reduction of the animal weight [77].

Inspired in combretastatin A-4, a small library of β-Lactams was explored as MTAs [78]. All compounds showed potent growth inhibitory effect against combretastatin A-4 sensitive MCF-7 human breast cancer and combretastatin A-4 resistant HT-29 colon cancer cells, with IC_50_ values in the nanomolar range. Among them, 3-hydroxy-β lactam **7** (Figure 5) demonstrated improved activity over combrestatatin A-4 in combrestatatin A-4 resistant HT-29 colon cancer cells. The comparison of the results obtained in the screening assays allowed the SAR considerations highlighted in Figure 5. Compounds **7**–**10** revealed to inhibit tubulin polymerization, being compound **7** the most potent inhibitor (5.4-fold reduction in Vmax [maximum rate of reaction], as combretastatin A-4). Further studies concluded that **7** could interact with CBS and was a potent inhibitor of tubulin assembly at micromolar concentrations. [78].

Jian et al. (2020) synthesized a library of novel pyrazolo [3,4-b] pyridine-bridged analogs of combretastatin A-4 and evaluated their biological activity [79]. Firstly, these compounds were tested for their antiproliferative activity against MCF-7, MDA-MB-231, HeLa, and esophageal squamous cell carcinoma (Kyse150) cell lines. The results of this assay highlighted analog **11** (Figure 5) as the most potent, with IC_50_ values in the low micromolar range. SAR studies indicated that the group of synthesized compounds with a 3,4,5-trimethoxyphenyl at C-3 of the pyrazolo [3,4-b] pyridine ring possessed weaker antiproliferative activity than the group substituted by a 3,4,5-trimethoxyphenyl group on the C-1. Within this more promising group, it was found that compound **11** substituted with a 3-hydroxy-4-methoxyphenyl group at C-3 possessed a stronger antiproliferative activity than the other analogs possessing at the same position a phenyl group substituted by electron-withdrawing groups, such as fluorine, chlorine, carbomethoxy and cyano in the *para* position (Figure 5). Further in vitro assays allowed to verify that **11** promoted cycle arrest at the G2/M cell phase, and inhibited tubulin polymerization. Molecular modeling studies suggested the interaction of **11** with CBS as combretastatin A-4 [79].

AQ-4 (**12,** Figure 5), a 4-arylaminoquinazoline structurally related to verubulin, a well-known microtubule destabilizer, was synthesized and evaluated for its effect on microtubules by Lin et al. (2020) [80]. In vitro antiproliferative studies demonstrated that **12** exhibited promising antiproliferative activity, with IC_50_ values ranging between 8 and 30 nM in a diverse panel of human cancer cell lines, derived from lung, breast, liver, stomach, colon, prostate, and ovary. Additionally, **12** showed potent activity against cisplatin, paclitaxel and vincrisitne drug-resistant cancer cells while exhibiting no toxicity towards normal cells. Further target validation studies, using A549 lung cancer cells, confirmed that **12** interacted with the tubulin-microtubule system, effectively inhibiting tubulin polymerization and disrupting intracellular microtubule dynamics. Competitive inhibition analysis confirmed that **12** bound to the CBS, with an inhibition rate of 81.8% at 10 μM. Consequently, **12** induced cell cycle arrest at the G2/M phase, promoted apoptosis (increase in cleaved poly (ADP-ribose) polymerase (PARP) and caspase-3 activation by 3.2-fold), and leaded to the collapse of mitochondrial membrane potential. The significant anticancer effects observed in vivo in mouse xenograft models, evidenced by areduction in tumor weight, underscore the therapeutic potential of **12** and warrant further investigation to fully assess its applicability in cancer treatment [80].

From the screening of a small library from the Korea Chemicals Banks by Han et al. (2020), the novel 5-(3-chlorophenyl)-N-(3-pyridinyl)-2-furamide (CPPF, **13,** Figure 5) was identified as a potential anticancer agent [81]. CPPF revealed potent antiproliferative effect on a panel of cancer cell lines, with IC_50_ values in the nanomolar. The anticancer effect of **13** was further supported by its ability to inhibit growth in 3D tumor spheroid cultures [81]. The induction of mitotic arrest was evidenced by the increase of the levels of mitotic markers PLK1, cyclin B1, and CDC25C. Molecular modeling and a competitive binding assay suggested the binding of **13** to the CBS. Noteworthy, the antitumor potential of **13** was maintained in adriamycin-resistant MCF-7 (MCF-7/ADR) and adriamycin-resistant human lymphoblastoid (K562/ADR) cancer cell lines and showed low toxicity against zebrafish models at 10 μM, causing no zebrafish death, morphological changes, or significant neurotoxicity. Its in vivo anticancer potential was validated using in vivo xenograft mouse model and a two-step skin cancer mouse model [81].

**Figure 6:**
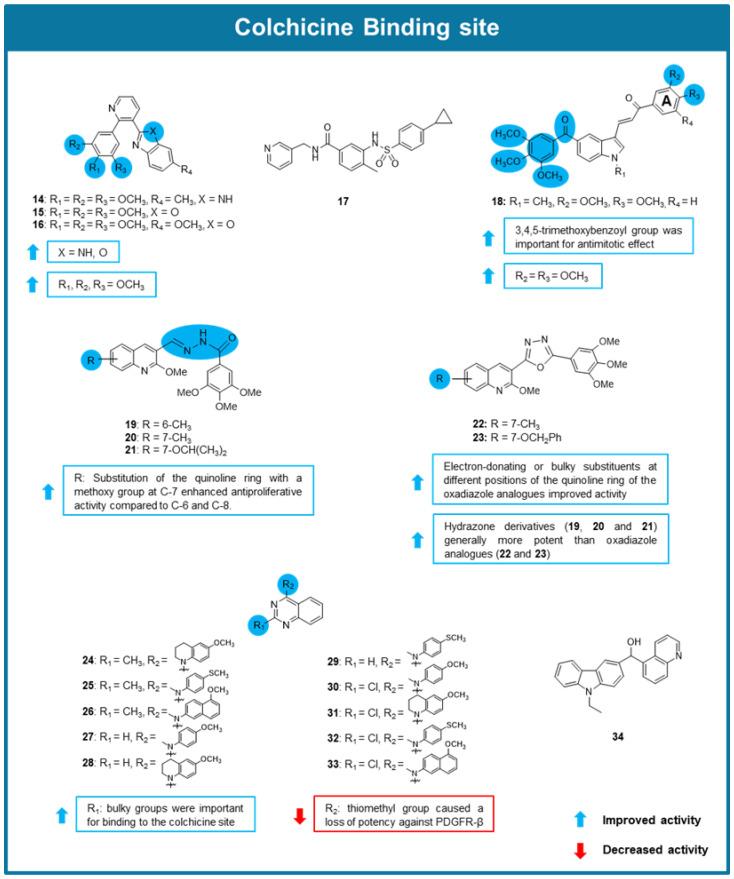
MTAs **14**–**34** with anticancer properties targeting the CBS.

Inspired by the antimitotic effect of combretastatin A-4 and several analogs with heterocyclic scaffolds, a series of arylpyridine derivatives were tested for their anticancer potential by He et al. (2020) [82]. From this series, compounds **14** and **16** (Figure 6) were found to significantly inhibit tubulin polymerization, being compound **16** (IC_50_ value of 2.1 μM), a 2-trimethoxyphenylpyridine bearing benzo[d]imidazole and benzo[d]oxazole side chains, the most promising. Some SAR considerations were reported as highlighted in Figure 6. An immunofluorescence assay was later conducted on A549 cells, where it was observed that **16** disrupted microtubule networks resulting in their disassembly and fragmentation. Moreover, compound **16** caused G2/M cell cycle arrest of A549 cells. To elucidate the potential binding site of compound **16**, molecular docking simulations were performed, which suggested its interaction with the CBS, like combretastatin A-4 [82].

Du et al. (2020) tested the antitumor activity of S-40 (**17,** Figure 6), a new tubulin destabilizer characterized by a benzamide core bounded to pyridine and benzene sulfonamide moieties [83]. X-ray analysis of **17** in a complex with tubulin revealed that S-40 occupies all three zones in the colchicine pocket with interactions that differ from known microtubule inhibitors, displaying distinct effects on the assembly and curvature of tubulin dimers. Compound **17** showed exceptional cytotoxicity against multiple cancer cell lines (IC_50_ values ranging from 0.006 to 0.062 μM), blocking mitosis and inhibiting the growth of a prostate cancer 3D in vitro tumor model (IC_50_ value of 0.046 μM). Interestingly, its antiproliferative activity was maintained in drug-resistant breast carcinoma (MX-1R) and drug-resistant A549 (A549R) cell lines and in an organoid derived from lung cancer (IC_50_ = 0.077–0.142 μM). To characterize the effects of **17** on tubulin assembly, prostate cancer (DU145) cells treated with this compound were observed by confocal microscopy. In this assay, compound **17** induced a reduction and disruption of microtubule networks, as well as cell shrinkage. Afterward, observations in cancer cells treated with compound **17** revealed an accumulation of cyclin B1, along with apoptosis markers such as cleaved caspase-3 and PARP. Besides these markers, a significant increase of the annexin V-positive population in annexin V staining assay was notedin DU145 and non-small cell lung carcinoma (NCI–H1299) cell lines. These results indicate that this compound blocks mitosis and induces apoptosis. A wound-healing assay was also conducted in DU145 and NCI–H1299 cancer cells which showed the inhibitory effects of **17** on cancer cell migration. Additionally, a colony formation assay demonstrated that even at a low concentration of 0.1 μM, **17** reduced cancer cell colony formation. This compound was also tested in a xenograft mouse model to determine its pharmacokinetic properties. In this assay, **17** demonstrated fast absorption and a bioavailability value of 11.1% at an oral dose of 10 mg/kg. Further research was done in DU145 and non-small cell lung cancer (H1299) xenograft mouse models, where exposure to **17** at 20 mg/kg led to tumor growth inhibition of 72.2 and 68.7%, respectively. Additionally, this compound was able to maintain its in vivo antitumor activity in an A549R xenograft mouse model, despite resistance mechanisms. Moreover, no neurotoxicity effects were observed in PC12 cells treated with **17**, in contrast to cells treated with paclitaxel and vinorelbine. Overall, **17** demonstrated promising anticancer activity overcoming paclitaxel resistance and lacks neurotoxicity, which are the main obstacles limiting clinical applications of paclitaxel. However, it demonstrated some pharmacokinetic limitations due to its metabolic instability [83].

From the screening of a library of new phenstatin-based indole-linked chalcones possessing a 3,4,5-trimethoxybenzoyl group and different substituents in the chalcone A aromatic ring, compound **18** (Figure 6), with a 2,3-dimethoxyphenyl A ring emerged as the most promising inhibitor of the growth of oral cancer (SCC-29B) cell line (GI_50_ < 0.1 μM) [84]. The presence of a 2,3-dimethoxy substitution on the A ring enhanced activity and the presence of a 3,4,5-trimethoxybenzoyl group was shown to be essential for inhibiting tubulin polymerization (Figure 6). Compound **18** also caused effective growth inhibition of oral squamous cancer spheroids cells, as well as bulk oral cancer cells. An immunofluorescence assay showed that the treatment of SCC-29B cells with 2.5 µM of **18** caused loss of polygonal shape, cell rounding, nuclear degradation, and apoptotic blebbing. To evaluate the in vivo antitumor activity of **18**, an oral squamous cell carcinoma (AW13516) xenograft mouse model was used. Compound **18** was administered intravenously, causing significant tumor reduction and presenting a tumor growth inhibition (T/C %) of 0.4. Furthermore, tumor sections demonstrated that angiogenesis was significantly reduced with increased necrosis and did not show any signs of toxicity. Masson’s trichrome staining revealed cell body shrinkages, shortening of cell migratory processes, and diffused microtubule protein staining. Tubulin polymerization assay confirmed the direct interaction of compound **18** with tubulin, and a [^18^F]-fluorodeoxyglucose uptake study verified that the treatment of cancer cells with **18** decreases glucose uptake, indicating that **18** reduces proliferation and glycolysis metabolism of cancer cells in oral cancer xenograft mouse model. Molecular docking studies suggested that compound **18** exerted its effects because of its interaction with the CBS [84]. Interestingly, the 3,4,5-trimethoxybenzoyl ring in **18**′s structure adopted an orientation very similar to that of the 3,4,5-trimethoxyphenyl group of colchicine in the crystallized structure, reinforcing the importance of this group for the interaction with the CBS [84].

Ibrahim et al. (2020) synthesized and tested the antitumor potential of a series of quinoline containing combretastatin A-4 analogs [85]. All 20 synthesized compounds showed promising antiproliferative activity against MCF-7, leukemia (HL-60), HCT-116, and HeLa cell lines (IC_50_ values between 0.012–4.21 µM). All compounds displayed tubulin polymerization inhibitory effect (IC_50_ values between 1.32–35.64 µM), being confirmed that quinoline combretastatin A-4 analogs **19**–**23** directly bind to the CBS, using a [^3^H] colchicine binding assay. Some SAR considerations were established as highlighted in Figure 6. Amongst the derivatives, compound **21** was the most potent, showing antiproliferative activity with IC_50_ values ranging from 0.04 to 0.022 μM, and selectivity for cancer cells when compared to non-tumorigenic epithelial (MCF-10A) cell line (IC_50_ > 35 μM). Moreover, MCF-7 cells treated with **21** showed G2/M cell cycle arrest and induction of apoptosis [85].

Inspired by the efficacy of quinazoline-based compounds like verubulin acting as MTAs previously reported, three series of quinazoline derivatives with different substituents at the 2- and 4-positions of the quinazoline scaffold were prepared and explored for their anticancer potential [86]. Compounds **24**–**33** (Figure 6) showed potent antiproliferative effects in MDA-MB-435 melanoma cells (IC_50_ values between 0.0006–0.0124 µM), and displayed the ability to depolymerize microtubules in A-10 cells. Additionally, these compounds displayed remarkable antiproliferative activity in parental HeLa, β-III overexpressing HeLa, SK-OV-3 and SK-OV-3-MDR-1–6/6 cell lines, overcoming common resistance mechanisms. In a tubulin polymerization assay, compounds **25**, **26**, and **30** also displayed tubulin polymerization inhibitory effect, with IC_50_ between values of 0.47 and 0.64 µM. To confirm that the effects of these compounds on tubulin polymerization occurred through binding to the CBS, a competitive binding assay was performed, which validated this hypothesis. SAR considerations allowed to conclude that the substitution at the 2-position by small hydrophobic groups (CH_3_ and Cl) could contribute to the antimitotic effect, suggesting that a bulk substituent at the 2-position of the quinazoline scaffold could be important for binding to the CBS (Figure 6) [86].

The new brain penetrant MTA named ST-401 (**34**, Figure 6) developed by Horne et al. (2020) was tested for its antiproliferative activity in a NCI-60 cell line screening, achieving IC_50_ values between 0.023–0.069 µM in a panel of glioma cell lines [87]. Further testing against PD-glioma isolates in culture supported **34** antiproliferative potency. Cell viability measurements in MGG8 and T98G cell lines treated with **34** showed a decrease in cell viability (IC_50_ values of 0.014 and 0.036 µM, respectively). This compound was also capable of inhibiting tubulin polymerization (IC_50_ of 1.1 μM) and microtubule assembly in HCT-116 cells at 0.2 µM. It was verified that these effects result from its interaction with the CBS using a competition assay (89% inhibition of colchicine binding). The presence of compound **34** in brain tissue, alongside plasma levels, demonstrated its ability to penetrate the blood-brain barrier. In vivo antitumor activity was then tested in a COLO205 xenograft mouse model **34** (20 mg/kg, i.p.). Results showed that, tumor growth reduced to 200–250% compared to the growth of 400–550% in vehicle control. This dosage of compound **34** also showed no signs of toxicity in treated mice. Finally, this compound was also capable of enhancing temozolide and radiation therapy effectiveness [87].

**Figure 7:**
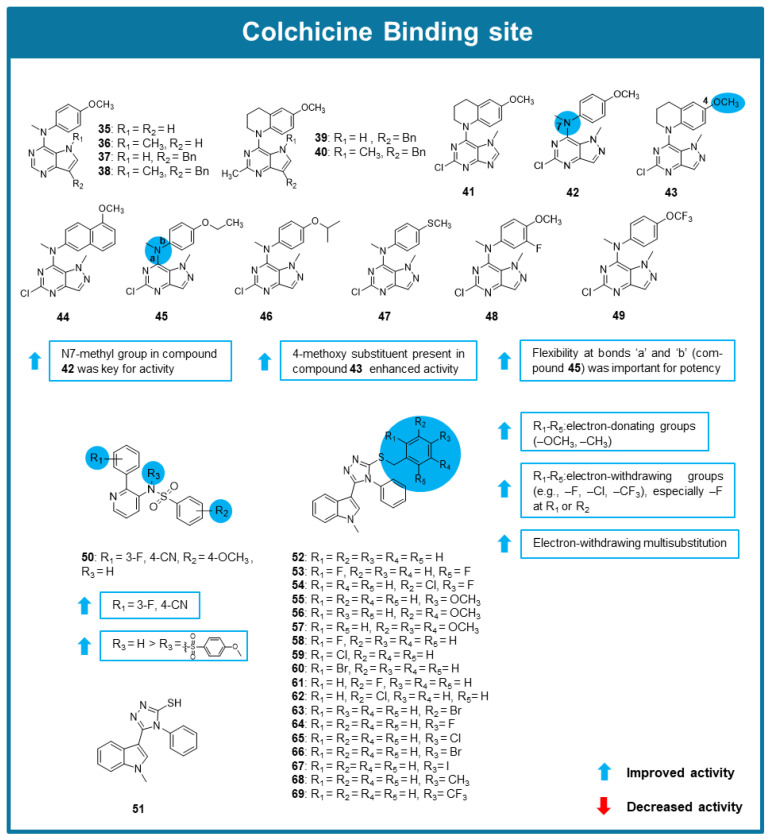
MTAs **35**–**69** with anticancer properties targeting the CBS.

A series of novel *N*1-methyl pyrazolo [4,3-d] pyrimidines (**35**–**49**, Figure 7) targeting the CBS were described by Islam et al. (2021) [88]. Compounds **42**–**45** behaved as potent inhibitors of tubulin polymerization, with IC_50_ values between 0.42–0.49 μM. The comparison of the results of the inhibition of colchicine binding assays allowed the SAR considerations reported in Figure 7. These compounds also inhibited [^3^H] colchicine binding to tubulin, with inhibition percentages between 87 and and 92% for compounds **42**–**45**, indicating a strong affinity for the CBS, comparable to combretastatin A-4. In vitro cytotoxicity studies, assessed using the sulforhodamine B (SRB) assay, demonstrated that compounds **42**, **44**, **45**, and **48** effectively inhibited the growth of MCF-7 breast cancer cells, with GI_50_ values in the range of 0.0175–0.007 μM. Notably, these compounds were also effective against βIII-tubulin overexpressing (*TUBB3*) MCF-7 cells, overcoming a key resistance mechanism to taxanes and other MTAs. Furthermore, compounds **42**–**49** circumvented P-gp-mediated drug resistance, as confirmed by cytotoxicity assays in NCI/ADR-RES ovarian cancer cells, which overexpressed P-gp. In a mouse xenograft model of MCF-7 *TUBB3* tumors, compound **42** significantly outperformed paclitaxel, reducing tumor volume by 79% whereas paclitaxel only achieved a 42% reduction under similar conditions. Importantly, no significant weight loss or signs of toxicity were observed in treated mice, highlighting the potential safety advantage of this compound. Collectively, these studies support the further preclinical development of the pyrazolo [4,3-d]pyrimidine scaffold as a new generation of tubulin inhibitors. Particularly, compound **42** exhibited superior potency compared to paclitaxel, both in vitro and in vivo, making it a promising candidate for overcoming drug resistance in cancer therapy [88].

From the search for new potential antitumor agents by Zhu et al. (2021), a new 2-aryl-3-sulfonamido-pyridine (HoAn32, **50**, Figure 7) was identified as a potential MTA [89]. Compound **50** exhibited potent antiproliferative activity against a panel of cancer cell lines, namely breast, liver, lung, colon, and pancreatic cancer, with IC_50_ values ranging from 1.193 to 0.170 μM. Antiproliferative evaluation allowed SAR considerations displayed in Figure 7. Compound **50** showed to inhibit the tubulin polymerization almost completely at 10 μM. The *N,N’*-ethylene-bis(iodoacetamide) competition assay in the colorectal carcinoma (SW620) cell line confirmed that **50** bound to the CBS, being these results in accordance with molecular modeling studies. Moreover, **50** promoted G2/M cell cycle arrest in RKO, SW620, and human pancreatic cancer (PANC-1) cell lines and progressive reduction in the expression levels of cyclin B1 and cdc2 kinase, and increased apoptosis in both RKO and SW620 cells at low concentrations (<1 µM). After exposure to 0.5 μM of **50**, an increase in reactive oxygen species (ROS) levels was observed in both RKO and SW620 cells. To evaluate this compound’s effects on cell migration, a wound-healing assay was performed in the umbilical vein endothelial (HUVEC) cell line after treatment with **50**, resulting in disturbance of HUVEC migration. In vivo testing of **50** in SW620 xenograft mouse models revealed that **50** reduced tumor growth by 53.6% with no apparent toxicity [89].

Wu et al. (2021) synthesized and evaluated the anticancer activity of new indole-1,2,4-triazole derivatives [90]. Compounds **51**–**69** (Figure 7) showed strong antiproliferative activity against HepG2, HeLa, MCF-7, and A549 cells (IC_50_ values between 0.15–13.13 μM), being capable of inhibiting tubulin polymerization (IC_50_ values ranging from 2.10 to 35.79 μM). Notably, SAR studies suggested that the presence of a phenyl group connected to the 1,2,4-triazole ring and its substitution with electron-donating groups (such as OCH_3_) were associated with an improvement of the antiproliferative activity (Figure 7). The most promising of this series was compound **57**, being its antiproliferative activity associated with apoptotic cell death, G2/M cell cycle arrest and a decrease in the levels of anti-apoptotic proteins. A tubulin polymerization assay supported the idea that this compound promotesdsignificant inhibitory activity (IC_50_ value of 2.1 μM). To determine toxicity against non-cancer cell lines, the HEK-293T cell line was treated with compound **57** and underwent an MTT assay, where compound **57** demonstrated low toxicity (IC_50_ of 126 μM). The molecular docking of compound **57** in tubulin suggested its interaction with the CBS [90].

**Figure 8:**
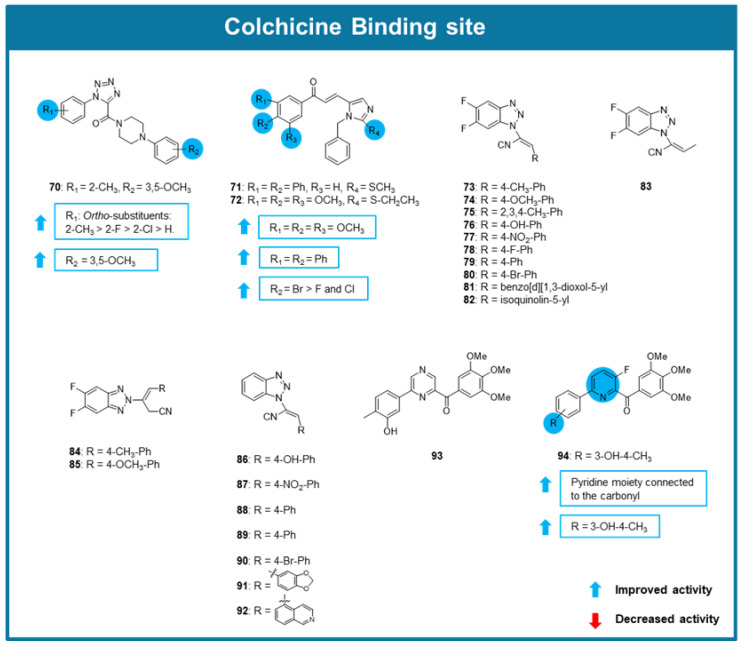
MTAs **70**–**94** with anticancer properties targeting the CBS.

A series of 1-aryl-5-(4-arylpiperazine-1-carbonyl)-1*H*-tetrazols with tubulin polymerization inhibitory effect was explored by Wang et al. (2021) [91]. Amongst the tested compounds, **70** showed the most promising antiproliferative activity against gastric adenocarcinoma (SGC-7901), A549, and HeLa cancer cell lines (IC_50_ between 0.09 and 0.268 μM). The comparison of the results in the screening assays suggested that the presence of a substituent at the *ortho* position of the phenyl group connected to the tetrazole ring enhanced antiproliferative activity. The most favorable substituent at this position was a 2-methyl group (2-methyl>2-fluoro>2-chloro>H), which is present in the most active compound (compound **70**). The presence of a 3,5-dimethoxyphenyl group connected to the piperazine is also associated with an enhancement of the activity (Figure 8). Compound **70** hindered tubulin polymerization and caused microtubule disorganization in SGC-7901 cells by binding to tubulin. Additionally, compound **70** inhibited SGC-7901 cells in the G2/M phase. Molecular modeling suggested that these analogs bound to the CBS [91].

In 2021 two new imidazole-chalcone derivatives (**71, 72**, Figure 8) with potential antimitotic effects were discovered by Oskuei et al. (2021) [92]. These compounds showed promising antiproliferative activity against A549, MCF-7, mitoxantrone resistant MCF-7 (MCF-7/MX), and HepG2 cell lines, being compound **72** the most active with IC_50_ values between 7.05 and 21.97 μM. Both compounds triggered G2/M cell cycle arrest and increased the number of apoptotic cells in A549 cells. As combretastatin A-4, **71** and **72** also inhibited tubulin polymerization. Molecular docking suggested that this compound exerted its effects due to interactions with the CBS. Interestingly, once again, the compounds showing the most promising activity presented a 3,4,5-trimethoxyphenyl group as combretastatin A-4 [92].

From a study of Riu et al. (2021), a new series of 5′,6′-difluorobenzotriazole-acrylonitrile derivatives (**73**–**92**) as MTAs has emerged [93]. Docking studies predicted the binding of these compounds with the CBS on tubulin. The antiproliferative activity against a panel of 60 cancer cell lines confirmed that 1*H*-benzotriazole (**73**–**83**) were more potent than 2*H*-benzotriazole (**84** and **85**) derivatives (Figure 8). Among the tested compounds, **73** exhibited the highest cytotoxicity activity, with an IC_50_ of 3.2 μM in HeLa cells. Cell cycle analysis by flow cytometry showed G2/M phase arrest in Hela cells treated with **73**, indicating that **73** disrupts mitotic progression. Additionally, immunofluorescence staining for tubulin and DNA, confirmed that **73** induced microtubule depolymerization, consistent with its role as a microtubule-destabilizing agent. Competitive colchicine-binding assays showed that **73** competes with colchicine, further validating its tubulin-targeting mechanism. Moreover, co-administration of **73** with an efflux pump inhibitor significantly increased its intracellular retention, suggesting that **73** is a substrate for efflux transporters. These findings highlighted compound **73** as a promising anticancer agent, particularly in combination therapies targeting drug resistance mechanisms. Nevertheless, further in vivo studies will be required to confirm its therapeutic potential [93].

A series of 6-aryl-2-benzoyl-pyridines were developed and tested for their anticancer activity against a panel of breast cancer and melanoma cell lines [94]. Among these compounds, **93** and **94** (Figure 8) displayed potent antiproliferative activity with average IC_50_ values of 0.0092 and 0.0024 µM, respectively. Compound **94** was found to strongly inhibit tubulin polymerization, and crystallographic analyses proved the interaction of these compounds with the CBS. Besides this, **94** was also able to maintain its antiproliferative activity against multidrug-resistant cell lines. In a clonogenic assay, **94** reduced colony formation of A375/TxR cells. Additional testing showed that **94** induced G2/M cell cycle arrest and increased the proportion of apoptotic cells in A375/TxR cells. In vivo testing using A375/TxR xenograft model demonstrated that **94** achieved a tumor growth inhibitory rate of 44.4%. The staining with hematoxylin/eosin (H&E) revealed that tumors treated with **94** were highly necrotic and significantly less metastatic [94].

**Figure 9:**
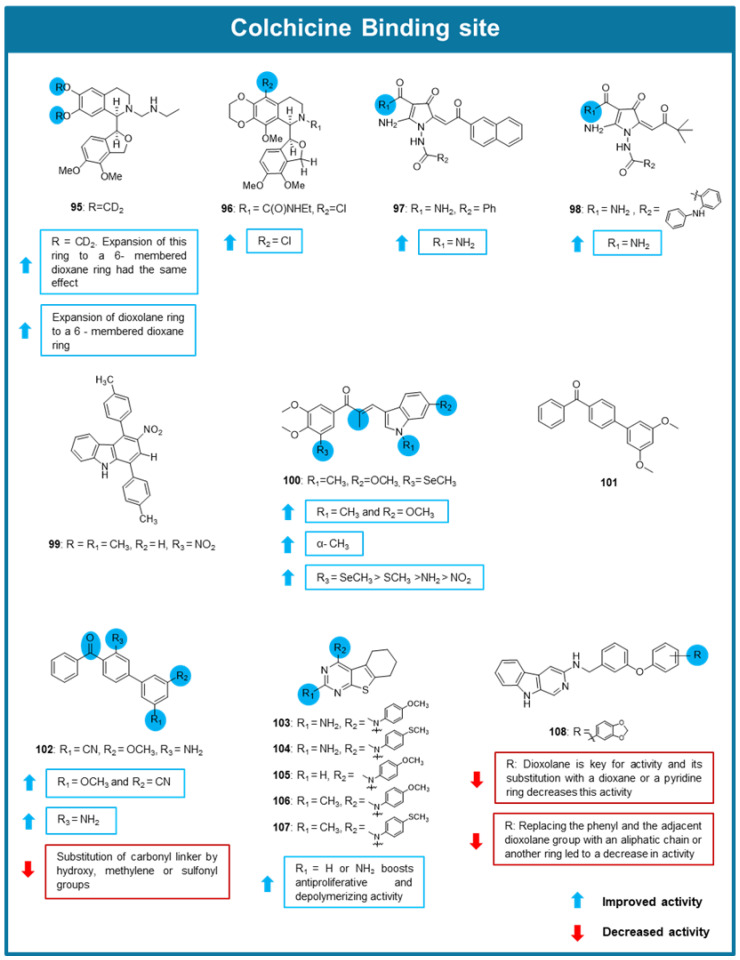
MTAs **95**–**108** with anticancer properties targeting the CBS.

In 2021, two 1,3-benzodioxole-modified noscapine analogs (**95** and **96**, Figure 9) were reported for their promising antimitotic effect [95]. The deuterated noscapine derivative **95** and the dioxino-containing analog **96** showed potent cytotoxic effects against breast MCF-7 cells with EC_50_ values of 1.5 and 0.73 μM, respectively. Compound **96** also displayed promising cytotoxic activity against a panel of 60 cancer cell lines from melanoma, non-small cell lung carcinoma, brain, kidney and breast cancers, with EC_50_ values lower than 2 µM. Notably, compound **96** demonstrated promising efficacy against taxane-resistant cancer cell lines, where the EC_50_ values were significantly lower than those for the taxane-resistant controls, suggesting a robust ability to bypass MDR. In these resistant models, compound **96** showed an EC_50_ of 0.81 μM in drug resistant breast cancer cells (NCI^ADR/RES^). Furthermore, X-Ray crystallography techniques were employed to confirm the binding of **95** and **96** to the CBS. Both compounds showed to effectively inhibit tubulin polymerization in a tubulin polymerization assay [95].

Two new pyrrole-based carboxamides (**97** and **98**, Figure 9) with potent anticancer properties against several epithelial cancer cell lines, including MDA-MB-231 breast, NSCLC lung, and PC-3 prostate cancer were reported from Boichuk et al. (2021) [96]. Both compounds showed ability to interfere with the microtubule network and inhibit tubulin polymerization, being this effect predicted to be associated with the binding to the CBS on the tubulin by molecular docking studies. Furthermore, these compounds induced G_2_/M phase arrest in HCC1806 breast cancer cells (~50–60%), as assessed by flow cytometry. Both compounds induced apoptosis, as evidenced by a significant increase in apoptotic markers. In a HCC1806 breast cancer xenograft model, **97**- and **98**-treatment resulted in a significant reduction in tumor size, confirming their anticancer efficacy in vivo [96].

Carbazole derivatives have been largely studied for their anticancer activity through the interaction with several targets, namely microtubules. In this respect, Sinicropi et al. (2021) have described a series of eight carbazole derivatives with promising antitumor effect [76]. Among them, compound **99** (Figure 9) exhibited potent antiproliferative activity against MCF-7 and MDA-MB-231 cancer cells, and displayed moderate toxicity against normal human mammary epithelial cells MCF-10A. Docking studies suggested its interaction with the CBS [76].

Another series of chalcone derivatives with an indole moiety as tubulin polymerization inhibitors was designed by Yan et al. in 2022 [97]. Antiproliferative testing of these derivatives was conducted against A549, HeLa, hepatocellular carcinoma (MHCC-97H), colorectal carcinoma (HCT-8), ovarian cancer (A2780), and MCF-7 cell lines [97]. Amongst the tested chalcones, compound **100** was the most potent, showing IC_50_ values between 0.006 and 0.035 μM. Noteworthy, the evaluation of the cytotoxic effect of **100** towards human normal cell lines, three drug-resistant cell lines (MCF-7/ADR, HCT-8/VCR, and A2780/TAX) revealed that **100** had significantly higher cytotoxic activity against human cancer cells than human normal cells, with this cytotoxic effect being maintained in cancer cells resistant to adriamycin, vincristine and paclitaxel. In this study, various SAR considerations were proposed as highlighted in Figure 9. Mechanistic studies allowed to verify that **100** inhibited tubulin polymerization (IC_50_ value of 0.81 μM) and significantly promoted disturbance and shrinking of microtubules. A colchicine competition binding assay further revealed the strong binding of **100** to the CBS. Moreover, promotion of G2/M cell cycle arrest, apoptosis, and inhibition of TrxR (IC_50_ = 10.24 μM), resulting in the accumulation of ROS and superoxide anions were detected. The antitumor potent activity of **100** was also tested in an in vivo cell line-derived xenograft mouse model. In this model, **100** caused suppression of tumor growth, with no detectable reduction in body weight, indicating a favorable safety profile [97].

From the exploration of the antitumor properties of a small library of unsymmetric biphenyl derivatives, compound **101** was identified as the most potent with an IC_50_ value of 0.094 µM, being this effect associated with the inhibition of tubulin polymerization [98]. The EBI competition assay allowed to conclude that dxy-1-175 interact with the CBS. Further molecular optimization of **101** resulted in the synthesis of different 4-benzoylbiphenyl analogs with modifications at A, B, C rings and the linker which were tested for their in vitro growth inhibitory effect against a panel of cancer cells, including leukemia (RAW264.7, HL60 and THP1), cervical cancer (Hela), fibrosarcoma (HT1080) and breast cancer (4T1). From the results, some SAR considerations were obtained as highlighted in Figure 9. Compound **102** exhibited the strongest antiproliferative effect, with IC_50_ values in the nanomolar range against the tested cancer cell lines, while lacking tissue-specific selectivity among these tumor types. Notably, **102** demonstrated high selectivity towards cancer cells over normal human lung fibroblasts (MRC-9). Importantly, **102** effectively overcame P-gp-mediated MDR and may not be a substrate of P-gp, indicating that **102** could be valuable in treating drug-resistant tumors. At 30 µM, **102** inhibited tubulin polymerization by 75.0%, correlating with its antiproliferative activity. An EBI competitive binding assay confirmed that **102** interact with the CBS. Furthermore, **102** induced significant G_2_/M cell cycle arrest. In a murine 4T1 breast cancer xenograft model, **102** significantly inhibited tumor growth, underscoring its potential as an antitumor agent [98].

A series of 4-substituted 5,6,7,8-tetrahydrobenzo [4,5]thieno [2,3-d]pyrimidines targeting the CBS of tubulin were designed, synthesized and tested for their antimitotic effects by Islam et al. (2022) [99]. Compounds **103**–**107** (Figure 9) displayed significant antiproliferative effect against MDB-435 cells (IC_50_ = 0.009–0.088 µM), inhibited tubulin assembly (IC_50_ = 0.49–2.3 µM) better than combretastatin A-4 (IC_50_ = 1 µM), and inhibited the binding of [^3^H] colchicine to tubulin by 89–99%. Compounds **103**–**107** were tested for their ability to overcome βIII-tubulin-mediated drug resistance using an isogenic pair of HeLa cell lines [99], being **103** the most potent. Rr values (~1.0) obtained for compounds **103**–**107** suggested that they circumvent βIII-tubulin mediated drug resistance, in contrast to paclitaxel. Compounds **103**–**107** were also evaluated for their activity in SK-OV-3 ovarian carcinoma and its P-gp-expressing subline. All compounds exhibited low Rr values (between 1–1.5), suggesting that they are poor P-gp substrates with advantages over taxanes and vinca alkaloids in MDR cancer cells, with **103** showing the best results. Compound **103** was further assessed in the NCI-60 human cancer cell line panel, where it exhibited potent antiproliferative activity. In vivo efficacy studies of compound **103** using a MDA-MB-435 xenograft model allowed to verify that **103** promoted a moderate body weight loss but achieved statistically significant tumor growth inhibition [99].

From the antitumor screening of a library of 3-amino-β-carboline derivatives by Li et al. (2022), compound **108** (Figure 9) demonstrated remarkable antiproliferative activity against a panel of cancer cells (IC_50_ values between 0.005 and 0.014 µM) [100]. A competitive binding assay with colchicine in HeLa cells revealed that **108** promoted α,β-tubulin degradation by directly binding to the CBS. It was further demonstrated that when HeLa cells were pretreated with 0.010 µM of a proteasome inhibitor (MG-132), **108** could not induce the degradation of α,β-tubulin, indicating its effects on the ubiquitin-proteasome pathway. Noteworthy, the potent antiproliferative activity of this compound was maintained in MDR cancer cells, namely paclitaxel-resistant ovarian (A2780T) and lung cancer (A549T) cells, as well as adriamycin-resistant human breast, and P-gp-overexpressed MCF-7 (MCF-7/AD) cancer cell lines. Immunofluorescence assay performed in A2780S cells treated with 0.01 µM of **108** revealed a promotion of the inhibition of microtubule polymerization and spindle formation. Moreover, treatment of HUVEC cells with **108** caused inhibition of cell migration, and this compound also induced G2/M cell cycle arrest and apoptosis in A2780S and A2780T cell lines. Pharmacokinetic properties were also tested in mice, indicating an acceptable oral bioavailability of 30.70%. In A2780S and A2780T xenograft mouse models, **108** displayed significant in vivo tumor growth inhibition, with no apparent toxicity [100].

**Figure 10:**
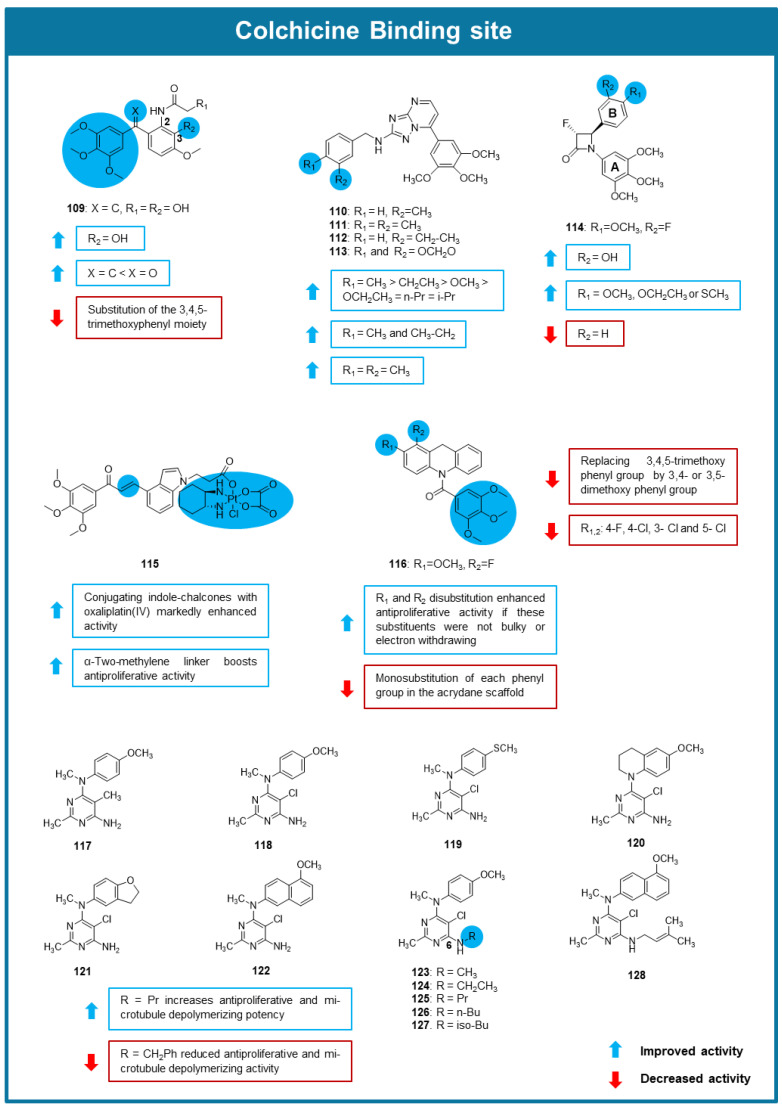
MTAs **109**–**128** with anticancer properties targeting the CBS.

Zhou et al. (2022) synthesized and evaluated the antitumor activity of a series of benzophenone, stilbene, and phenstatin derivatives [101]. Antiproliferative activity testing against A549, HeLa, A2780, HCT- 8, and MCF-7 cell lines highlighted the phenstatin derivative **109** as the most promising, displaying IC_50_ values between 0.01 and 0.05 µM. The effect of **109** on the in vitro growth of other cell lines, including non-tumorigenic cell lines and drug-resistant cell lines, proved that it exhibited selectivity for cancer cell lines and that it maintained efficacy on drug-resistant cell lines. SAR analysis of the phenstatin analogs showed that the presence of a vinylic group in the linker between the two benzene rings is associated with a more potent antiproliferative effect than a carbonyl group. The presence of a 3,4,5-trimethoxyphenyl group is favorable for the antiproliferative activity and the substitution of this group by a heterocyclic ring led to a decrease in the antiproliferative activity. Moreover, the substitution of the other aromatic ring at C-2 by an amide group and at C-3 by a hydroxy group was beneficial for the activity (Figure 10). Compound **109** showed potent tubulin polymerization inhibitory activity with an IC_50_ value of 0.58 µM, and this effect was attributed to its binding to the CBS. Moreover, **109** induced G2/M cell cycle arrest in A549 cells and promoted disruption of the microtubule network. Although it acted as the MTA, compound **109** triggered cell death mainly through cell ferroptosis rather than apoptosis. Pharmacokinetic studies demonstrated the favorable oral (69.45%) bioavailability of compound **109** and its fast absorption. A549 xenograft mouse models were used to evaluate compound **109**′s antitumor activity. In this assay, treatment with 10 mg/kg of **109** induced a tumor growth inhibition of 78.63% compared to the positive control erastin which caused only 10.90% inhibition [101].

The potential antimitotic effect of a library of new 2-anilino triazolopyrimidines was explored by Romagnoli et al. (2022) [102]. Compounds **110**–**113** showed the most potent antiproliferative activity on MDA-MB-231, HeLa, A549, and HT-29 cancer cells. The comparison of the results of the antiproliferative activity allowed some SAR considerations highlighted in Figure 10. The study of the mechanism of action of these compounds allowed to conclude that **110**–**113** caused inhibition of tubulin polymerization through the interaction with the CBS, being **110** the most potent (IC_50_ value of 0.45 μM in tubulin polymerization assay, 72% inhibition in colchicine binding assay). Compound **110** was also shown to promote G2/M cell cycle arrest, and apoptosis accompanied by a decrease in mitochondrial potential in HeLa cells. To evaluate the in vivo toxicity of compound **110**, zebrafish embryos were exposed to a concentration of 0.3 μM. This dose was well tolerated, with no observed significant adverse effects. In vivo zebrafish models injected with DiI-labeled HeLa cells and subsequently treated with compound **110** (0.3 μM) exhibited reduced tumor cell dissemination [102].

A series of 3-fluoro and 3,3-difluoro substituted β-lactams analogs of combretastatin A-4 were synthesized and evaluated for their antitumor activity by Malebari et al. (2022) [103]. From the group of synthesized compounds, **114** (Figure 10) showed low toxicity in non-cancer cells (HEK-293T cells), and significant cytotoxic effect against MCF-7 cancer cells (IC_50_ value of 0.095 µM). In addition, **114** proved to be effective against MDA-MB-231 cancer cells with an IC_50_ value of 0.62 µM. Compound **114** was also selected for additional testing in NCI-60 cell line screening at 10 µM, with the most potent growth inhibition being observed in the leukemia panel. Mechanistic studies show that **114** caused apoptosis in MCF-7 cells, and inhibited tubulin polymerization. In silico studies indicated the interaction of the compound with the CBS [103].

To overcome drug resistance and reduce the side effects of oxaliplatin (OXA) in colon cancer treatment, six platinum(IV) complexes with indole–chalcone derivatives were synthesized by Cao et al. (2022), and were tested for their antiproliferative effects on various colon cancer cell lines [104]. Among them, indole–chalcone oxaliplatin (IV)-propanoate (**115**, Figure 10, which featured a two-methylene linker and was derived from oxaliplatin (IV), demonstrated the most potent anticancer activity (IC_50_ ranging from 0.13 to 0.43 μM). It showed superior effects compared to monotherapy or combinations in both parent and OXA-resistant HCT-116 (HCT-116/OXA) cells, with notable selectivity for cancer cell lines, and significantly inhibited HCT-116 cell migration. Immunofluorescence analysis and docking studies also showed that **115** can bind to the CBS, thereby preventing tubulin polymerization. This enhanced efficacy was linked to a substantial increase in intracellular drug accumulation and improved DNA damage. Mechanistic studies revealed that **115** increased ROS accumulation, and activated the mitochondrial apoptotic pathway [104].

Peng et al. (2023) designed, synthesized and evaluated the biological activity of a new series of acridine-based tubulin polymerization inhibitors [105]. The in vitro antiproliferative testing against HCT-116, murine melanoma (B16-F10), HeLa and HepG2 cell lines showed that most compounds displayed a high growth inhibitory effect, dependent on the substitution pattern of the aromatic rings (Figure 10). Among them, compounds with a 3,4,5-trimethoxybenzoyl group showed to be particularly active, being **116** the most potent, reaching IC_50_ values between 0.02 and 0.041 µM, lower than colchicine. Further studies allowed to conclude that this compound behaved as a tubulin polymerization inhibitor (IC_50_ = 1.5 μM) and promoted both G2/M cell cycle arrest and apoptosis in B16-F10 cells. Molecular modeling further revealed that **116** bound directly to the CBS when interacting with tubulin. Using the melanoma B16-F10 tumor model, **116** was evaluated for its in vivo antitumor activity and in vivo immunomodulatory effects when administered in monotherapy or with a PD-L1 inhibitor. Results showed that **116** (10 mg/kg) caused no significant weight loss, indicating a good safety profile and displayed a tumor growth inhibition value of 65.1%, which was raised to 77.6% when combined with PD-L1 inhibitor (10mg/kg) [105].

Aiming to optimize the anticancer activity of a previously identified microtubule depolymerizing agent targeting the CBS with a pyrrolo [2,3–d] pyrimidine scaffold, a series of monocyclic pyrimidine analogs were synthesized, and evaluated for their antitumor activity by Choudhary et al. (2023) [106]. Compounds **117**–**128** inhibited the growth of MDA-MB-435 cancer cells and promoted cellular microtubule depolymerization in A-10 cells, being this effect associated with the inhibition of colchicine binding (% inhibition with 5 µM inhibitor between 45 and 84%). Amongst the obtained compounds, **125** was shown to be 47-fold more potent (EC_50_ = 0.123 µM) for cellular microtubule depolymerization activity and 7.5-fold more potent (IC_50_ = 0.0244 µM) at inhibiting the growth of MDA-MB-435 cancer cells compared to the lead compound. SAR studies suggested that the presence of a propyl group linked to the amine at C-6 of the pyrimidine was associated with improved activity (Figure 10). Molecular docking studies suggested that **125** binded to the CBS. This compound was also able to overcome βIII tubulin-mediated drug resistance and was not affected by P-gp expression. In vivo, testing with an MDA-MB-435 xenograft mouse model revealed that treatment with **125** did not demonstrate statistically significant antitumor activity, but showed a trend towards lower tumor volume [106].

**Figure 11:**
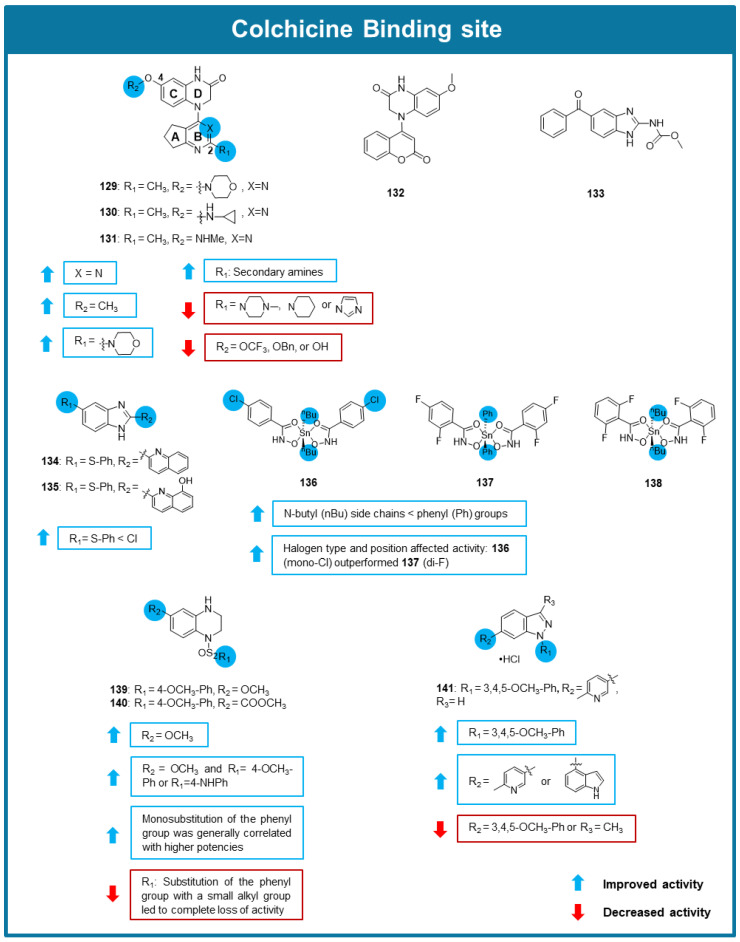
MTAs **129**–**141** with anticancer properties targeting the CBS.

Inspired by the potent antimitotic effect of a previously discovered tetrahydroquinoline quinazoline, Pochampally et al. (2023) designed and synthesized a library of dyhidroquinoxalinone derivatives and tested its antiproliferative activity against cancer cells [107]. Among tested compounds, **129**–**131** exhibited the strongest antiproliferative activities against various cell lines, including melanoma, breast, pancreatic, and prostate cancer cells, displaying IC_50_ values between 0.0002 and 0.0562 µM. SAR studies indicated that the presence of amino groups at C-2 in the pyrimidine moiety and of a methoxy group linked to the C-4 of the phenyl group are beneficial for the activity (Figure 11). X-Ray crystallographic analysis allowed to conclude that compounds **129**–**131** bound to the CBS, with the A and B rings adjacent to the β-tubulin and the C and D rings adjacent to the α-tubulin. The interaction with tubulin in the CBS of compound **131** was confirmed by EBI competition assay in PC-3 cells, and by tubulin polymerization assay, confirming its action as a destabilizing agent. Interestingly, this compound presented good stability and a longer half-life than its parental compound in liver mouse microsomes and was selective to cancer cells when compared to a monkey fibroblast-like (COS-7) cell line. Cell cycle analysis showed that **131** caused G2/M arrest in PC-3 cells. The wound-healing assay confirmed the capacity of **131** to inhibit cell migration, displaying complete inhibition of wound-healing at 0.005 µM. In vivo testing of **131** using a PC-3/TxR xenograft mouse model showed strong tumor growth inhibition and angiogenesis inhibition at the well-tolerated dose of 2.5 mg/kg [107].

Song et al. (2023) synthesized a new coumarin dihydroquinoxalone derivative, MY-673 (**132**), and tested its anticancer potential using in vitro and in vivo assays [108]. The structure of this derivative consisted of a chroman-2-one group bounded to a 7-methoxy-3,4-dihydroquinoxalin-2(1*H*)-one group (Figure 11). Docking studies suggested that both these moieties form hydrogen bonds and hydrophobic interactions with the CBS. **132**′s anticancer activity was substantiated by its antiproliferative activity against multiple breast, prostate, lung, gastric and esophageal cancer cell lines with an average IC_50_ value of 0.0212 µM. This derivative was found to act on multiple cellular pathways, inhibiting ERK1/2 proteins, RSK1 (regulated by ERK), and TGF-β/SMAD signaling pathway. It was also observed by flow cytometry analysis that **132** induced G2/M phase arrest of MGC-803 and HGC-27 cell lines at 0.020 µM. This compound was also capable of inhibiting gastric cancer cell colony formation and migration, down-regulating the expression levels of the cell migration-related proteins MMP2 and MMP9. These inhibitory properties of compound **132** were found to be dependent on the ERK signaling pathway. Observation of apoptosis-like changes in gastric cancer cells revealed that **132** caused the downregulation of anti-apoptotic proteins (Bcl-2, Mcl-1, and c-IAP1), upregulation of pro-apoptotic protein Noxa, and significant cleavage of caspase-3/9 as well as PARP, indicating that this compound induced apoptosis. To verify in vivo antitumor activity, **132** was tested at 20 mg/kg dose on a mice-bearing MGC-803 cells xenograft model [108]. This compound caused significant tumor growth inhibition and was well tolerated, being confirmed in this model that it effectively induced cancer cell apoptosis [108].

A study developed by Graff et al. (2023) showed that methyl *N*-(6-benzoyl-1*H*-benzimidazol-2-yl)carbamate (**133**, Figure 11), an anthelmintic microtubule disruptor, bound to the CBS and can be effective against MTA-resistant metastatic breast cancer [109]. Compound **133** significantly affected various cellular processes, including clonogenic survival, cell cycle, apoptosis, autophagy, and mitotic catastrophe. The effect of **133** on cell viability was tested in various breast cancer and normal cell lines. The **133** treatment inhibited clonogenic viability in all breast cancer cell lines, with EC_50_ values ranging from 162 nM to 637 nM. Interestingly, breast cancer cells with mutant p53 were more sensitive to **133** than those with wild-type p53. In contrast, compound **133** exhibited over 10-fold greater potency in breast cancer cells compared to normal mammary epithelial cells, suggesting a broader therapeutic window relative to docetaxel and vincristine. These findings suggest that **133** could be a promising new MTA for treating metastatic breast cancer [109].

From the exploration of the antitumor properties of a small library of benzimidazole derivatives, compounds **134** and **135** (Figure 11) emerged as the most potent growth inhibitors of two cell lines from lung and breast cancer (IC_50_ values between 2.33 and 7.17 µM). [110]. Additionally, compounds **134** and **135** demonstrated selectivity indexes of 5.81 and 5.20, respectively, using lung normal (MRC-5) cell lines. Both compounds inhibited cancer cell motility and migration and induced early-phase apoptosis in A549 cells. They were also found to have microtubule-stabilizing properties. The results from in vitro tubulin polymerization and immunofluorescence assays, and docking studies suggested that **134** and **135** exerted their anticancer effects by stabilizing the microtubule network through interaction with the CBS [110].

Song et al. (2023) reported the synthesis and anticancer activity evaluation of organotin benzohydroxamate derivatives [111]. Compounds **136** and **137** (Figure 11) revealed the most potent antiproliferative activity against six human cancer cell lines (HeLa, EC, HepG2, HEC, T24, and SHSY-5Y). In vivo study of compound **136** demonstrated significant tumor weight reduction in H22 and S180 models, with tumor inhibitory ratios of 53.91% and 46.25%, respectively, and low toxicity. Results from the EB1 competition assay indicated that compound **136** exerted its antitumor effects through direct binding to the CBS, a mechanism further supported by molecular docking studies, which predicted interactions of this compound as well as of compounds **137** and **138** with the CBS. This study provides strong evidence supporting the antitumor effects of organotin benzohydroxamate derivatives, which were attributed to the direct binding to β-tubulin at the CBS, disruption of the microtubule network, mitotic arrest at the metaphase/anaphase junction, and the subsequent induction of apoptosis [111].

Dong et al. (2023) synthesized and evaluated the antiproliferative activity towards human colon cancer cells HT-29 of a series of tetrahydroquinoxaline sulfonamide derivatives [112]. Compounds **139** and **140** (Figure 11), which exhibited the most potent antiproliferative activity in preliminary screening, were further evaluated in HT-29, HepG2, HeLa, and MCF-7 cell lines, with compound **139** showing the highest potency (IC_50_ values ranging from 2.2 to 7.52 µM). Both compounds caused disrupted microtubule morphology in HT-29 cells, and **139** was shown to inhibit tubulin polymerization. Treatment of HeLa cells with 4 µM of **139** promoted G2/M cell cycle arrest, but no significant signs of apoptosis induction were observed. Molecular docking studies indicated the potential of compound **139** to interact with the CBS [112].

From the screening of a newly synthesized series of water-soluble indazole derivatives by Cui et al. (2023), compound **141** (Figure 11) emerged as the most promising in vitro growth inhibitor of a panel of four cancer cell lines, including A549 (IC_50_ = 0.027 μM), human hepatocellular carcinoma (Huh-7, IC_50_ = 0.03 μM), urinary bladder carcinoma (T24, IC_50_ = 0.046 μM), and taxol-resistant A549 (A549/Tax, IC_50_ = 0.033 μM) cell lines [113]. SAR studies indicated that the indazole core should be substituted with a 3,4,5-trimethoxyphenyl moiety at the NH position to obtain the most potent antiproliferative activity [113]. Amongst the derivatives possessing this substitution, the hydrochloride salt with a 6-methylpyridin-3-yl substitution in the indazole core (compound **141**) displayed the best antiproliferative activity. Compound **141** inhibited tubulin polymerization (IC_50_ = 1.75 μM) and inhibited microtubule aggregation as well as spindle formation in A549 cells. The interaction of **141** with the CBS was confirmed through an EBI competition assay. Further analysis showed that after treatment with 0.0125 μM of compound **141**, A549 cells accumulated in the G2/M phase. In addition to its effects on the cell cycle of A549 cells, treatment with compound **141** at 0.0125 μM resulted in a significant increase in apoptosis and inhibition of cell migration. In vivo, antitumor efficacy was confirmed using A549 xenograft mouse models that demonstrated a significant reduction in tumor volume (64.1%) at 25 mg/ kg, with no signs of toxicity [113].

**Figure 12:**
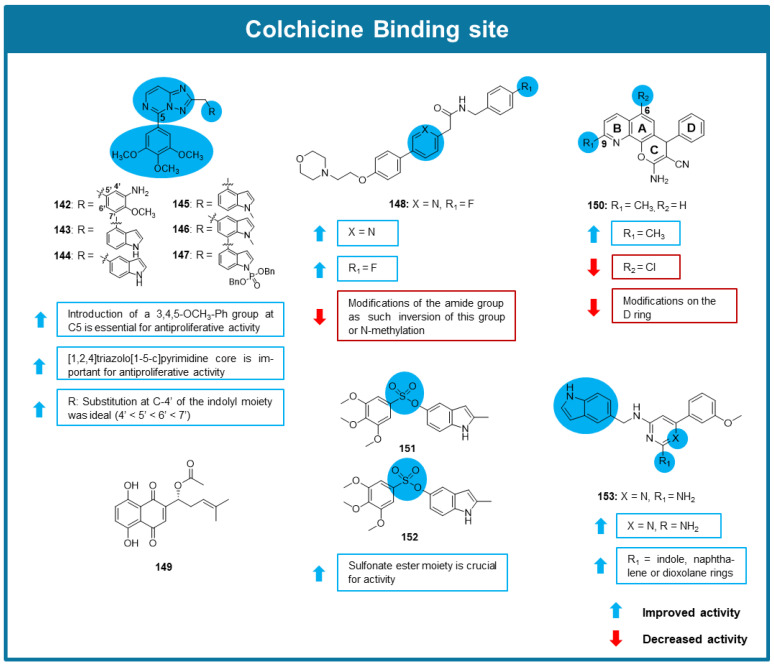
MTAs **142**–**153** with anticancer properties targeting the CBS.

In a study by Chen et al. (2023), a novel series of substituted 2-amino [1,2,4]triazolopyrimidines was synthesized and evaluated as potential dual inhibitors of microtubule polymerization and Janus Kinase 2 (JAK2) [114]. Antiproliferative screening, along with JAK2 expression analysis in a panel of five cancer cells, identified compounds **142**–**147** (Figure 12) as the most promising (IC_50_ values ranging from 0.009 to 1.8 μM). Molecular docking studies suggested the interaction of all compounds with CBS, and in vitro tubulin polymerization assays confirmed their inhibitory effect on tubulin polymerization (IC_50_ values of 1.3–15.3 μM). In addition, **142**–**146** exhibited strong inhibitory activity against JAK2. Among them, **143** demonstrated cancer cell selectivity, the ability to induce G2/M cell cycle arrest, and apoptosis in A549 cells. Additionally, low resistance indices (0.3–0.36) were observed when tested against drug-resistant cell lines (A2780/TAX, HCT-8/VCR, MCF-7/ADR, and A549/DDP) and their parental counterparts. Considering the poor water solubility and limited oral bioavailability of **143**, its phosphate sodium salt (**147**, Figure 12) was prepared, giving rise to a prodrug with improved water solubility and enhanced oral bioavailability. Finally, in an A549 xenograft mouse model, treatment with **143** and **147** at 50 mg/kg resulted in tumor weight reductions of 70% and 91%, respectively, compared to the control group [114].

Inspired by tirnabulin, an MTA with a dual mechanism of action combining Src signaling inhibition and disruption of microtubule dynamics, Park et al. (2023) synthesized a library of tirnabulin analogs and evaluated their anticancer potential [115]. The comparison of the antiproliferative activity of synthesized compounds allowed some SAR considerations, as highlighted in Figure 12. The most promising analog was compound **148**, displaying an IC_50_ value of 0.04 µM against the HeLa cell line. Molecular docking studies suggested that this compound bound to the CBS, similarly to tirnabulin. Through cell cycle analysis, it was observed that this analoginduced G2/M cell cycle arrest in HeLa cells, and tubulin polymerization assay confirmed that compound **148** targeted microtubules and disrupted tubulin polymerization. Similarly to tirnabulin, compound **148** at 0.050 µM also inhibited the tyrosine-protein kinase Src signaling pathway in HeLa cells. Additionally, compound **148**′s biocompatibility was evaluated through in vitro cytotoxicity assays using nontumorigenic cell lines treated with concentrations of **148** below 4 µM. Compound **148** demonstrated no significant cytotoxic effects and unaltered cell morphologies, suggesting its safety [115]. In vitro ADME studies made it possible to conclude that **148** showed good microsomal stability in both mice and humans and a percentage of plasma protein binding within acceptable ranges. Animal pharmacokinetics studies demonstrated excellent oral bioavailability [115].

The exploration of bioactive natural products from *Lithospermum erythrorhizon* resulted in the identification of acetylshikonin (**149**, Figure 12), with promising antiproliferative activity against a panel of cancer cell lines from lung, cervical, prostate, liver, and intestinal cancers (IC_50_ = 1.09–7.26 μM). [116]. Noteworthy, a reduced cytotoxicity towards normal cells (L-02 and RWPE-1) and a maintenance of the antiproliferative activity against drug-resistant cells (MHCC-97H/CDDP, PC-3/ENZR, and HCT-8/VCR) were observed, demonstrating the selectivity of **149** towards cancer cells and the activity in drug-resistant cells [116]. Compound **149** also revealed the capacity to inhibit tubulin polymerization (IC_50_ value of 5.98 μM) and disrupt the morphology of microtubules characterized by the disappearance of filamentous structures, shrinkage, and punctate distribution. The interaction site of **149** on tubulin was determined to be the CBS using a colchicine competitive binding assay, with the binding to this site found to be irreversible. The analysis of MHCC-97H cells exposed to 0.75 μM of **149** showed significant G2/M cell cycle arrest and the induction of apoptosis. Additionally, caspase-3 activity was significantly elevated in the **149**-treated group. A decrease in mitochondrial membrane potential, a sign of early cell apoptosis, was also observed [116].

Yang and colleagues (2024) designed and synthesized a series of novel 4-aryl-4*H*-chromene derivatives [117]. Compound **150** (Figure 12) showed promising antiproliferative activity against malignant glioma (U87), A549, and Huh7 cancer cell lines, with IC_50_ values between 0.88 and 1.19 µM. SAR analysis showed that substitution of C-9 with a methyl group led to an improvement in cytotoxic activity (Figure 12). In contrast, when C-6 was substituted with chlorine the cytotoxic activity decreased. Modifications on the D ring also had the same result. To clarify the molecular target of compound **150**, a transcriptome analysis based on the RNA sequences of U87 cells was conducted [117]. In this assay, it was observed that *TUBA1A* and *TUBA1B*, as well as β-subunit-related genes *TUBB* and *TUBB4B*, were downregulated by **150**, indicating that microtubules are a potential target for this compound. This was confirmed via tubulin polymerization assay. Moreover, molecular dynamic studies suggested the affinity of this compound to the CBS. Fluorescence staining conducted on U87 cells treated with **150** revealed that this compound degraded the intracellular tubulin skeletons. Further studies allowed researchers to conclude that the antiproliferative activity on U87 cells of **150** was related to the induction of G2/M cell cycle arrest and apoptosis. To evaluate the in vivo antitumor activity of compound **150,** mice bearing orthotropic glioma were treated with this compound (5 mg/kg and 10 mg/kg). This treatment resulted in the inhibition of tumor growth, with no apparent significant toxicity to the mice [117]. Hematoxylin and eosin staining, as well as immunohistochemistry, further confirmed these results and showed compound **150**′s capacity to lower tumor microvessel density. Finally, in silico simulation studies and P-gp transwell assays showed a positive correlation between compound **150**′s blood–brain barrier (BBB) permeability and its in vivo anti-glioblastoma activity [117].

Homer et al. (2024) reported a series of sulfonate ester analogs of combretastatin A-4 designed to target the CBS [118]. Among these, compounds **151** and **152** (Figure 12) exhibited exceptional antiproliferative activity in a panel of five cancer cell lines, with IC_50_ values ranging from 0.0037 to 0.0154 μM. When evaluated in a more physiologically relevant 3D model using a panel of patient-derived PDAC organoids, **151** and **152** retained their nanomolar-range efficacy. Mechanistic studies, including X-Ray crystallography and a competition assay using 2-methoxy-5-(2,3,4-trimethoxyphenyl)-2,4,6-cycloheptatrien-1-one (MTC), confirmed that their activity stemmed from binding to the CBS. Additionally, both compounds effectively induced cell cycle arrest in MIA PaCa-2 PDAC cells [118].

A series of 45 derivatives of the tubulin degradation agent BML284 were synthesized by Zhang et al. (2024) [119]. The most potent of these derivatives was **153** (Figure 12) demonstrating IC_50_ values between 0.02 and 0.05 µM when tested against HeLa, HCT-116, MCF-7, chronic myelogenous leukemia (K562), and acute monocytic leukemia (Molm 13) cell lines. Compound **153** caused tubulin degradation at a concentration as low as 1 μM, with this effect associated with the inhibition of tubulin polymerization through the interaction with the CBS. Moreover, **153** induced G2/M cell cycle arrest in A2780S and A2780T cells and apoptosis in A2780S cells. Noteworthy, the antiproliferative activity of **153** was maintained when tested in MDR cell lines, including βIII-tubulin-overexpressed (A2780T and A549T), and P-gp-overexpressed (MCF-7/ADR) cell lines, displaying IC_50_ values between 0.016 and 0.037 µM. In vivo antitumor activity of **153** was tested on A2780S and A2780T xenograft mouse models, with tumor growth inhibition rates reaching 45.3% on the A2780S model and 52.2% on the A2780T model at a dose of 10 mg/kg [119].

Doan et al. (2024) designed, synthesized, and evaluated the anticancer potential of new colchicine-combretastatin A-4 analogs containing quinoline [120]. These analogs were submitted to in vitro cytotoxicity testing against MDA-MB-231 cells. This assay identified compounds **154**–**157** as the most potent, reaching IC_50_ values between 14.54 and 34.97 µM. The comparison of the antiproliferative activities of the synthesized derivatives suggested that the substitution of the benzene ring in the *N*-((5,6,7-trimethoxyquinolin-3-yl) methyl) aniline structure with a 1*H*-indole-5-yl moiety, along with the presence of a methoxy group in the 2-position of the quinoline group, led to the best improvement in antiproliferative activity. In silico studies allowed the researchers to predict that compounds **154**–**157** possess adequate physicochemical features, pharmacokinetic profiles, and could bind to the CBS, like colchicine and combretastatin A-4. Nevertheless, their affinities for α,β-tubulin were less effective than those of colchicine and combretastatin A-4 [120].

**Figure 13:**
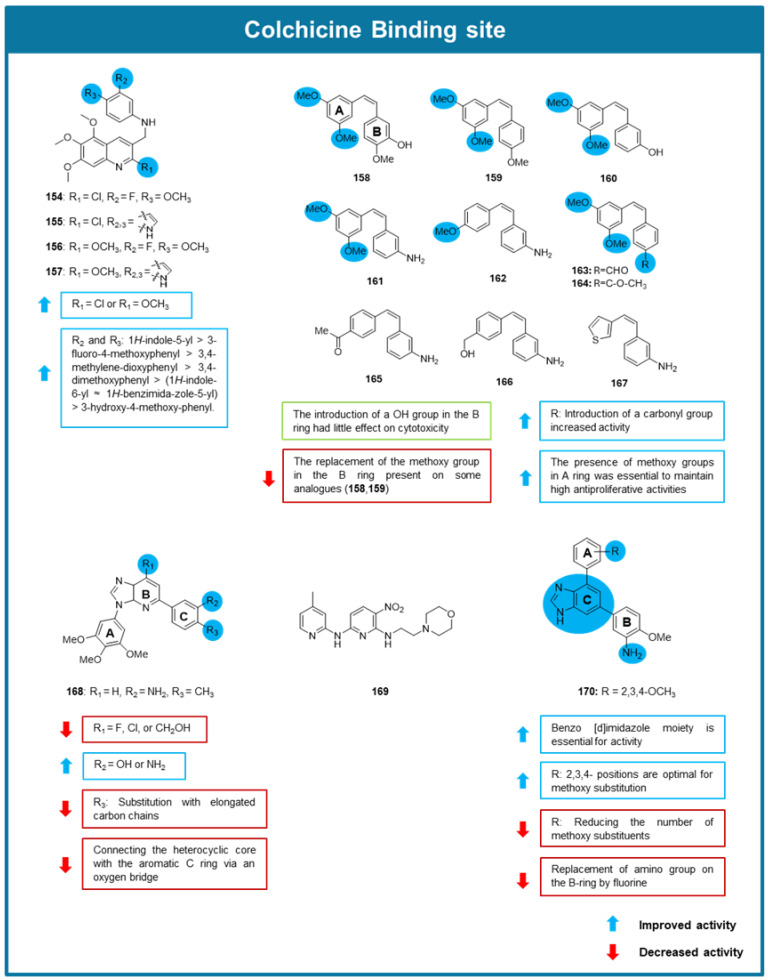
MTAs **154**–**170** with anticancer properties targeting the CBS.

In 2024, a series of combretastatin A-4 analogs targeting CBS (**158**–**167**) with potent antiproliferative activity against K562, HCT-116, HL-60, and H1299 cancer cells was identified, with compound **161** being the most potent (IC_50_ values of 0.53, 24.61, 0.35, and <0.3, respectively) [121]. SAR evaluation performed on the synthesized analogs concluded that both methoxy groups at positions 3 and 5 of the A ring were critical for cytotoxic activity and that the addition of a carbonyl group to the B ring, as observed in compound **161**, enhanced cytotoxic activity while a hydroxy substitution had only a slight effect (Figure 13) [121].

Jiang et al. (2024) designed, synthesized, and evaluated the biological activity of a novel series of diaryl-substituted fused heterocycles [122]. Amongst these compounds, **168** (Figure 13) showed the most potent antiproliferative activity against the MCF-7 cancer cell line (IC_50_ value of 0.0014 μM) and maintained this effect on drug-resistant cell lines (A2780/T, A549/T, A2780/CDDP, and A549/CDDP). Analysis of the mechanism of action of this compound revealed that its anticancer activity was dependent on its interaction with katanin as well as with the CBS in microtubules, inhibiting tubulin polymerization (IC_50_ value of 2 μM). An immunofluorescence assay revealed that MCF-7 cells treated with **168** showed disorganized fibrous microtubule structures and a decrease in density. This compound at 0.01 μM also induced G2/M cell cycle arrest and apoptosis in MCF-7 cells. The evaluation of the in vivo antitumor activity of **168** in an MDA-MB-231 xenograft mice model at 15 mg/kg, showed tumor weight reductions of 84%, with no significant signs of toxicity. H&E staining further revealed the development of necrosis in tumors treated with **168**, while tumors in the vehicle-treated group had no such development. A549/T xenograft model treatment with a dose of 15 mg/kg resulted in a 74% reduction in tumor weight, while all mice maintained their body weights. Evaluation of **168**′s pharmacokinetic properties determined that this compound had a favorable pharmacokinetic profile with an oral bioavailability of 40%. [122].

In a study by Herman et al. (2024), a series of 3-nitropyridine analogs were synthesized and evaluated for their antitumor activity [123]. Antiproliferative screening assays identified compound **169** (Figure 13) as highly potent against HT-29 colorectal cancer cells, with an IC_50_ value of 0.004 μM. Subsequent testing in the NCI-60 cancer cell line panel revealed average GI_50_ values of 0.0219 μM. Importantly, this compound exhibited minimal cytotoxicity towards peripheral blood mononuclear cells (PBMCs and MRC-5). Furthermore, in Jurkat T-cell leukemia cells, **169** induced apoptosis and G2/M cell cycle arrest. Concerning its mechanism of action, compound **169** induced disassembly of the microtubule network in A549 cells, demonstrating activity comparable to that of vinca alkaloids, and X-Ray crystallographic analysis indicated its interaction with the CBS. [123].

From the search for new diaryl-substituted fused nitrogen heterocycles with antitumor activity, compound **170** (Figure 13) emerged as the most promising in vitro growth inhibitor of A549 and drug-resistant A549/T cancer cell lines (IC_50_ values of 0.023 μM and 0.057 μM, respectively, and a resistance index of 2.47) [124]. Importantly, compound **170** demonstrated selectivity for cancer cells, showing minimal cytotoxicity in nontumorigenic MCF-10A cells. In vitro tubulin polymerization assays confirmed that compound **170** inhibited both the rate and extent of polymerization (IC_50_ = 1.8 μM). Consistent with these findings, A549 and A549/T cells treated with compound **170** (at 50 nM) displayed disrupted microtubule structures, and molecular modeling suggested its binding at the CBS. Additionally, **170** induced G2/M cell cycle arrest and apoptosis in both A549 and A549/T cells at 25 nM. Given its strong cytotoxicity against drug-resistant cells, its interaction with P-gp was assessed through flow cytometry and immunoblotting in A549/T cells, revealing that compound **170** was not a substrate of P-gp. Finally, in an A549/T xenograft mouse model, **170** administered at 15 mg/kg significantly suppressed tumor growth, outperforming the reference compound combretastatin A-4 [124].

### 6.4. MTAs Targeting Maytansine Binding Site

**Figure 14:**
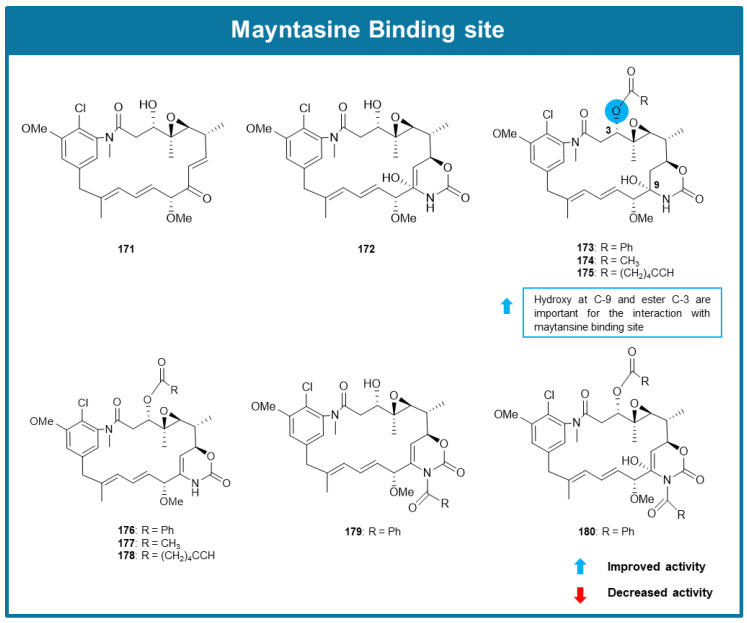
New MTAs with anticancer properties targeting the maytansine binding site.

A series of maytansine derivatives (**171**–**180**) were prepared by Marzullo et al. (2022) [125]. Compounds **173**–**177** and **179** behaved as strong inhibitors of tubulin assembly, while compounds **173**, **179**, and **181** displayed a mild inhibitory effect, and compound **171** revealed weak inhibition (Figure 14). Among these compounds, **175** (0.011 μM) and **175** (0.02 μM) showed the highest affinities for the maytansine binding site, with dissociation constants like the one of maytansine (0.014 μM), suggesting that the *N*-acetyl-*N*-methyl-l-alanine moiety present in maytansine can easily be replaced by other substituents. Nevertheless, compound **173** with a phenolic ester at position C-3 was a slightly weaker binder (0.051 μM). The compounds without the hydroxy group at C-9 showed binding affinities in the μM range, suggesting that the C-9-hydroxy group might be essential to allow establishing the interaction with the site. Compounds **178** (1.8 μM), **179** (3 μM), and **180** (2 μM), also not possessing the hydroxy group at C-9 and with modifications either at the C-3, the oxazinanone nitrogen, or both, displayed affinities in the sub-mM range. Amongst the obtained compounds, analog **173**, consisting of the maytansinol scaffold bounded to a phenyl group through an acyl moiety, was the most promising. This analog exhibited adequate binding affinity and remarkable cytotoxicity in several cancer cell lines, including adriamycin-resistant and P-gp-overexpressing A2780 (A2780AD) cell lines. Further studies were carried out with analog **173** through fluorescence microscopy using A549 cells. Observations after incubation of A549 cells with 0.01 μM concentration showed that **173** induced disorganization of the microtubule network, with signs of depolymerization during interphase in irregular bi-nucleated cells [125].

### 6.5. MTAs Targeting Laulimalide/Peloruside A Binding Site

**Figure 15:**
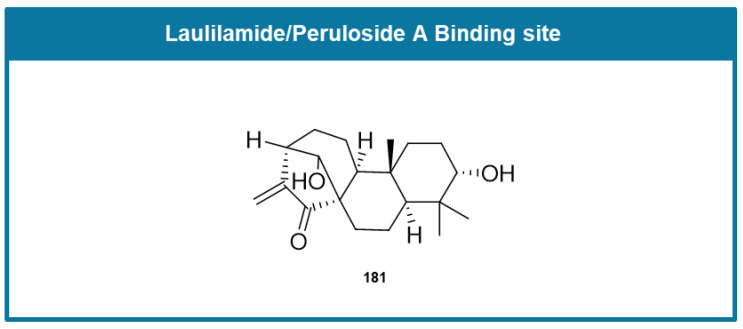
New MTAs with anticancer properties targeting the laulimalide/peloruside A binding site.

Yang et al. (2022) evaluated the anticancer potential of the entkaurane diterpenoid wangzaozin A (**181**, Figure 15), which was isolated from *Isodon racemosa* (Hemsl) Hara [126]. Compound **181** showed promising antiproliferative activity against HeLa and leukemia (HL-60) cell lines, with this effect associated with G2/M cell cycle arrest. The effect of **181** on microtubule structure was subsequently evaluated through the exposure of HeLa and HL-60 cells, resulting in an increase of oblate and lobulated nuclei in HeLa cells and of lobulated nuclei in HL-60 cells. The immunofluorescence technique in HeLa and HL-60 cells exposed that this compound allowed to verify that **181** caused an increase in the density of the intracellular microtubule network and initiated bundling of microtubules, evidencing the microtubule-stabilizing effect of **181**. This conclusion was further supported by the results obtained in the tubulin polymerization assay. Finally, molecular dynamic studies showed a high affinity of **181** for the taxane binding site and an even higher affinity for the laulimalide site, demonstrating a new binding mode distinct from other microtubule stabilizers [126].

## 7. Conclusions

In this article, 181 new MTAs targeting the seven well-established binding sites of MTAs reported in the last five years were reviewed. Although most of the compounds are synthetic, most of them have natural product scaffolds, reinforcing that nature continues to be an inspiration for the discovery of new MTAs.

The CBS was found to be the prime target of new recently discovered MTAs. On the contrary, binding sites, such as the gatorbulin and pironetin sites, have not yet had many advancements regarding the development of MTAs, but they remain as a possible alternative for further investigation. Noteworthy, the interaction with multiple binding sites has also been suggested by docking studies, namely, for compound **181**, which was found to interact with the taxane binding site and the laulimalide site, demonstrating a new binding mode distinct from other microtubule stabilizers. Nevertheless, this hypothesis should be confirmed by biological assays.

Most MTAs identified since 2020 act as microtubule destabilizers, with some also being reported as microtubule stabilizers. This difference can be explained by the fact that a vast majority of CBS inhibitors act as microtubules destabilizers. Nevertheless, some degradation agents were also identified (compounds **108**, **150**, and **153**), though their limited number can be attributed to the recent discovery of this class.

Concerning in vitro antiproliferative activity, we highlight compounds **94**, **108**, **130**, **131**, **168**, and **173** for maintaining remarkably low IC_50_ values (<0.1 µM) amongst a variety of cancer cell lines, showing selectivity for cancer cells. Specifically, compounds exhibited potent in vitro antiproliferative effects against drug-resistant cancer cell lines, with the most notable activity observed for compounds **94**, **108**, **168**, and **173**. Additionally, some of these compounds, namely, **94**, **108**, and **168**, further demonstrated significant in vivo activity in drug-resistant xenograft models and revealed both favorable bioavailability and a low toxicity profile, highlighting their therapeutic potential as new MTAs. Nevertheless, further in vivo studies, including ADME profiling, are urgently required for the most promising MTAs. Such investigations will not only confirm their efficacy in animal models but will also provide guidance for structural modification to increase pharmacological properties. Only via such thorough testing can we obtain these compounds from the laboratory to clinical trials, ultimately discovering novel, safe, and effective anticancer drugs.

## Data Availability

The data are contained within the article.

## Supplementary Materials

The following supporting information can be downloaded at https://www.mdpi.com/article/10.3390/molecules30163314/s1. Table S1: New microtubule targeting agents reported since 2020. References [73,74,75,76,77,78,79,80,81,82,83,84,85,86,87,88,89,90,91,92,93,94,95,96,97,98,99,100,101,102,103,104,105,106,107,108,109,110,111,112,113,114,115,116,117,118,119,120,121,122,123,124,125,126] are cited in the Supplementary Materials.

## Abbreviations

The following abbreviations are used in this manuscript:

MTA	Microtubule-targeting agent
SAR	Structure–activity relationship
MDR	Multidrug-resistant
GTP	Guanosine triphosphate
MAPs	Microtubule-associated proteins
MTOC	Microtubule-organizing center
CBS	Colchicine binding site
P-pg	P-glycoprotein
MRP	Multidrug resistance-associated proteins
EBI	N,N-ethylenebis(iodoacetamide)
