# Supplementary material for: Recent Advances in Microtubule Targeting Agents for Cancer Therapy
Source: Molecules. 2025 Aug 8;30(16):3314. doi: 10.3390/molecules30163314 (PMC12388312; doi:10.3390/molecules30163314)
Supplement: Supplementary file 1 [file molecules-30-03314-s001.zip › molecules-3755273-supplementary.pdf]

molecules

Review

Recent advances in microtubule targeting agents for cancer therapy

Henrique C. Assunção [1,2,†], Patrícia M. A. Silva [3,4,5,†], Hassan Bousbaa [5,*] and Honorina Cidade [1,2,5,*]

[1] Laboratory of Organic and Pharmaceutical Chemistry, Department of Chemical Sciences, Faculty of Pharmacy, University of Porto, Rua de Jorge de Viterbo Ferreira, 288, 4050-313 Porto, Portugal; henrique.c.a.117@gmail.com
[2] CIIMAR/CIMAR LA-Interdisciplinary Centre of Marine and Environmental Research, University of Porto, Terminal de Cruzeiros do Porto de Leixões, 4450-208 Matosinhos, Portugal
[3] Associate Laboratory i4HB-Institute for Health and Bioeconomy, University Institute of Health Sciences-CESPU, 4585-116 Gandra, Portugal; patricia.silva@cespu.pt
[4] UCIBIO-Applied Molecular Biosciences Unit, Translational Toxicology Research Laboratory, 1H-TOXRUN, IUCS-CESPU, University Institute of Health Sciences, 4585-116 Gandra, Portugal
[5] UNIPRO–Oral Pathology and Rehabilitation Research Unit, IUCS-CESPU, University Institute of Health Sciences, 4585-116 Gandra, Portugal
* Correspondence: hassan.bousbaa@iucs.cespu.pt (H.B.); hcidade@ff.up.pt (H.C.)
† These authors contributed equally to this work.

Table of contents:

Table S1. New microtubule targeting agents reported since 2020.

Table S1. New microtubule targeting agents reported since 2020.

Comp.	*In vitro* antiproliferative activity in screening assays	Other effects observed *in vitro* assays	*In vivo* assays: Antitumor effects and toxicity	ADME studies	Ref
		MTAs targeting taxane binding site			
**1**	MCF-7: IC_{50} = 0.0031 µM<br>MDA-MB-231: IC_{50} = 0.004 µM<br>BT474: IC_{50} = 0.0314 µM<br>BT549: IC_{50} = 0.0792 µM<br>MDA-MB-453: IC_{50} = 0.0031 µM<br>**MDR cancer cell lines:**<br>MDA-MB-231R: IC_{50} = 0.1385 µM<br>MCF-7R: IC_{50} = 0.0815 µM<br>**Non-cancer cell lines**:<br>293T: IC_{50} = 0.0063 µM<br>MCF-10A: IC_{50} = 0.0027 µM	**MDR cancer cell lines**:<br>- Promoted tubulin polymerization in MCF-7R and MDA-MB-231R cells at 0.01 µM.<br>- Induced G2/M cell cycle arrest in MCF-7R (0.005 µM), MDA-MB-231R (0.1 µM) as well as in their parental cell lines.<br>- Triggered apoptosis and endoplasmic reticulum stress in MCF-7R and MDA-MB-231R cells at 0.005 µM.	**MDR cancers**:<br>- Reduced tumor volume growth in MCF-7R and MDA-MB-231R xenograft mouse models at 10 mg/ kg.	ND	[1]
**2**	A549: IC_{50} = 5.99 µM<br>PC-3: IC_{50} = 4.17 µM<br>HepG2: IC_{50} = 3.36 µM<br>NRK-52E: IC_{50} = 6.99 µM	- Caused microtubular aggregation and wrinkling of the cell nucleus in HepG2 cells (3 µM).<br>- Stabilized tubulin assembly and promoted protofilament assembly at 20 µM.<br>- Increased G2/M phase arrest in HepG2 cells at 24 µM.<br>- Induced apoptotic effect in HepG2 cells at 6 µM.<br>- Decreased A549 cell migration at 3 µM.	ND	ADMETLab 2.0 predictions:<br>- predicted to have adequate absorption potency and an acceptable safety profile.<br>- complied with all drug-likeness properties.	[2]

		MTAs targeting vinca alkaloids binding site			
**3**	MDA-MB-231: IC_{50} = 0.47 µM<br>MDA-MB-468: IC_{50} = 0.06 µM<br>HeLa: IC_{50} = 0.05 µM<br>DLD-1: IC_{50} = 0.22 µM<br>HCT-116: IC_{50} = 0.1 µM<br>HH: IC_{50} = 0.08 µM<br>HuT78: IC_{50} = 0.09 µM<br>HL60: IC_{50} = 0.1 µM<br>KG-1: IC_{50} = 0.12 µM<br>MES-SA: IC_{50} = 0.13 µM<br>YCC3/7, YCC11: IC_{50} < 1 µM<br>**MDR cancer cell lines:**<br>MES-SA/Dx5: IC_{50} = 9.54 µM	- Inhibited tubulin polymerization at 10 µM.<br>- Induced G2/M cell cycle arrest in MDA-MB-231 cells at 2 µM.<br>- Induced apoptosis in MDA-MB-231 cells at 4 µM.<br>- Increased cellular levels of caspases-3 and -9 and reduced expression of anti-apoptotic proteins MCL-1 and Bcl-x, in MDA-MB-231 cells.<br>**MDR cancer cell lines:**<br>- Maintained antiproliferative activity in multidrug-resistant MES-SA/Dx5 cells comparable to the parental cell line.	ND	ND	[3]
**4**	MDA-MB-231: IC_{50} = 11.6 µM<br>MCF-7: IC_{50} = 7 µM<br>**Non-cancer cell lines**:<br>MCF-10A: IC_{50} > 200 µM	- Selectivity for cancer cells.<br>- Induced microtubule disorganization in MCF-7 cells at 7 µM.<br>- Triggered apoptosis in MCF-7 cells at 7 µM.	ND	ND	[4]
		MTAs targeting colchicine binding site			
**5, 6**	**5:**<br>A549: GI_{50} = 0.033 µM<br>HCT-116: GI_{50} = 0.015 µM<br>HepG2: GI_{50} = 0.027 µM<br>SW620: GI_{50} = 0.008 µM<br>HT29: GI_{50} = 0.0031 µM	**5**, **6**:<br>- **5** (IC_{50} = 3.39 µM) and **6** (IC_{50} = 4.77 µM) inhibited tubulin polymerization.<br>- Disrupted microtubule network in HCT116 cells at 0.02 µM.<br>- Promoted 81% (**5**) and 87% (**6**) G2/M cell cycle arrest in HCT116 cells at 0.02 µM.<br>- Triggered 17.4% (**5**) and 15.2% (**6**) accumulation of apoptotic HCT116 cells at 0.01 µM.	**5**,**6**:<br>- Reduced tumour growth by 45.3% (**5**) and 58.9% (**6**) in a HCT116 xenograft mouse model at an oral dose of 25 mg/kg.	ND	[5]

	**6:**<br>A549: GI_{50} = 0.065 µM<br>HCT-116: GI_{50} = 0.041 µM<br>HepG2: GI_{50} = 0.053 µM<br>SW620: GI_{50} = 0.024 µM<br>HT29: GI_{50} = 0.096 µM		- In a HCT116 xenograft mouse model at an oral dose of 25 mg/kg, both compounds caused no reduction in the animal weight.		
**7-10**	**7-10:**<br>CA-4 sensitive MCF-7: IC_{50} = 0.005-0.044 µM<br>CA-4 resistant HT-29: IC_{50} = 0.003-0.018 µM	**7-10:**<br>- Inhibited tubulin polymerization at 10 µM.<br>**7:**<br>- Induced G2/M phase arrest in MCF-7 and HT-29 cells at 1 µM.<br>- Downregulated the expression of anti-apoptotic proteins Mcl-1, Bcl-2 and survivin in MCF-7 cells at 0.1 µM.	ND	ND	[6]
**11**	MCF-7: IC_{50} = 27.22 µM<br>MDA-MB-231: IC_{50} = 27.04 µM<br>HeLa: IC_{50} = 18.08 µM<br>Kyse150: IC_{50} = 62.82 µM	- 31% inhibition of tubulin polymerization at 10 µM.<br>- Induced G2/M phase arrest in HeLa cells by26.93% at 9 µM.	ND	ND	[7]
**12**	Panel of human cancer cell lines, derived from lung, breast, liver, stomach, colon, prostate, and ovary:<br>IC_{50} = 0.008-0.03 µM<br>MR-90: IC_{50} = 0.326 µM<br>A2780: IC_{50} = 0.012 µM<br>HCT-8: IC_{50} = 0.019 µM<br>**<u>MDR cancer cell lines</u>**:<br>A549/CDPP: IC_{50} = 0.016 µM<br>A2780/PTX: IC_{50} = 0.015 µM<br>HCT-8/VCR: IC_{50} = 0.031 µM	- Inhibited tubulin polymerization by 88.9% at 10 µM.<br>- Disrupted intracellular microtubule dynamics in A549 cells at 0.001 µM.<br>- Induced cell cycle arrest at the G2/M phase in A549 cells (76.3% at 0.02 µM).<br>- Promoted apoptosis in A549 cells (80% at 0.02 µM).	- Reduced tumor weight in A549 xenograft tumor models by 71.7% at 30 mg/kg.	ND	[8]

	**Non-cancer cell lines**:<br>BJ: IC_{50} = 0.563 μM<br>NCM-460: IC_{50} = 0.614 μM				
**13**	Panel of cancer cell lines of cervical, breast, leukemia, hepatoma, stomach, lung, prostate and skin cancers: IC_{50} = 0.2 to 0.4 μM<br>K-562: IC_{50} = 0.386 μM<br>MCF-7: IC_{50} = 7.95 μM<br>**MDR cancer cell lines**:<br>MCF-7/ADR: IC_{50} = 1.91 μM<br>K562/ADR: IC_{50} = 0.326 μM	- Inhibited growth in 3D tumor spheroid cultures.<br>- Increased the number of apoptotic HeLa cells at 1 μM.<br>- Caused accumulation of PAPP, caspase-3, -7, -8, and - 9 at 10 μM.<br>- Increased the number of G2/M cells as well as the levels of mitotic markers PLK1, cyclin B1, and CDC25C, in HeLa cells at 1 μM.	- Low toxicity against zebrafish models at 10 μM, causing no zebrafish death, morphological changes or significant neurotoxicity.<br>- In a xenograft mouse model, it suppressed tumor size by approximately 20% at 50mg/kg.<br>- In a skin cancer model decreased the average number of tumors by 50% at 0.5 μM.	ND	[9]
**14-16**	**14**:<br>HT-29: GI_{50} = 2.9 μM<br>A549: GI_{50} = 2.8 μM<br>U251: GI_{50} = 1.7 μM<br>**15**:<br>HT-29: GI_{50} = 5.2 μM<br>A549: GI_{50} = 7.9 μM<br>U251: GI_{50} = 4.6 μM<br>**16**:<br>HT-29: GI_{50} = 2.1 μM<br>A549: GI_{50} = 0.89 μM<br>U251: GI_{50} = 0.27 μM	**- 14, 15,16:**<br>- Inhibited tubulin polymerization at 10 μM.<br>**16:**<br>- Inhibited tubulin polymerization (IC_{50} = 2.1 μM)<br>- Disrupted the microtubule network in A549 cells at 1 μM, resulting in its disassembly and fragmentation.<br>- Promoted G2/M cell cycle arrest in A549 cells at 1 μM.	ND	ND	[10]

**17**	DU145: IC_{50} = 0.062 μM<br>BGC823: IC_{50} = 0.051 μM<br>MCF-7: IC_{50} = 0.039 μM<br>HCT-116: IC_{50} = 0.023 μM<br>A549: IC_{50} = 0.053 μM<br>H1299: IC_{50} = 0.006 μM<br>MX-1: IC_{50} = 0.01 μM<br>**<u>MDR cancer cell lines</u>**:<br>MX-1R: IC_{50} = 0.142 μM<br>A549R: IC_{50} = 0.077 μM	- Showed antitumor efficacy on organoids derived from lung cancer (IC_{50} = 0.105 μM).<br>- Blocked mitosis and inhibited the growth of a prostate cancer 3D tumor model (IC_{50} = 0.046 μM).<br>- Disrupted and reduced microtubule networks and also induced cell shrinkage in DU145 cells at 0.1 μM.<br>- Upregulated Cyclin B1, cleaved caspase 3 and poly (ADP-ribose) polymerase in DU145 cells.<br>- Promoted DU145 (30.34%) and NCI–H1299 (40.2%) cell apoptosis at 0.03 μM.<br>- Inhibited cancer cell migration in DU145 and NCI-H1299 at 0.03 μM.<br>- Reduced cancer cell colony formation at 0.1 μM.<br>- No neurotoxicity effects in PC12 cells treated with 0.1 μM.	- Inhibited tumor growth by 72.2% and 68.7% in DU145 and H1299 xenograft mouse models, respectively, at 20 mg/kg.<br>**<u>MDR cancers</u>**:<br>- Maintained antitumor efficacy in A549R xenograft mouse models.	- Demonstrated a bioavailability of 11.1 % and fast absorption in xenograft mouse model at an oral dose of 10mg/kg.	[11]
**18**	SCC-29B: GI_{50} < 0.1<br>Hep-G2: GI_{50} > 100<br>HT-29: GI_{50} = 51 μM	- Inhibited the growth of oral squamous cancer spheroids cells (GI_{50} = 0.96 μM).<br>- Induced loss of polygonal shape, cell rounding, nuclear degradation, and apoptotic blebbing in SCC-29B cells at 2.5 μM.	- Tumor reduction (T/C % = 0.4) in AW13516 xenograft mouse model at 2.5 mg/kg.<br>- Reduced angiogenesis and increased necrosis in AW13516 xenograft mouse model.<br>- No toxicity in AW13516 xenograft mouse model.<br>- Decreased proliferation and glycolysis metabolism in AW13516 xenograft mouse model.<br>- Induced cell body shrinkages, shortening of cell migratory processes, and diffused microtubules.	ND	[12]

**19-23**	**19-23**<br>MCF-7: IC_{50} = 0.012-4.21 μM<br>HL-60: IC_{50} = 0.028-0.703 μM<br>HCT-116: IC_{50} = 0.024-3.83 μM<br>HeLa: IC_{50} = 0.028-1.151 μM<br>**21**:<br>**<u>Non-cancer cell lines</u>**:<br>MCF-10A: IC_{50} > 35 μM	**19-23**<br>- Tubulin polymerization inhibitory effect (IC_{50} = 35.64- 1.32 μM).<br>**21:**<br>- Selective towards cancer cells.<br>- Increased G2/M phase arrest in MCF-7 cells at 0.05 μM.<br>- Accumulation of apoptotic MCF-7 cells (12%) at 0.05 μM.	ND	ND	[13]
**24-33**	**24-33**:<br>MDA-MB-435: IC_{50} = 0.0006-0.0124 μM<br>HeLa: IC_{50} = 0.0006-0.0168 μM<br>SK-OV-3: IC_{50} = 0.0008-0.0181 μM<br>**<u>MDR cancer cell lines</u>**:<br>WT β-III HeLa cells: IC_{50} = 0.0006-0.0131<br>SK-OV- 3-MDR- 1–6/6: IC_{50} = 0.001-0.0234 μM	**24-33**:<br>- Depolymerization of microtubules in A-10 cells (EC_{50} = 0.0019-0.0234 μM).<br>**25, 26** and **30**:<br>- Inhibited tubulin polymerization (**25**: IC_{50} = 0.48 μM, **26**: IC_{50} = 0.64 μM, **30**: IC_{50} = 0.47 μM).	ND	ND	[14]
**34**	Panel of glioma cell lines (SF-268, SF-295, SF-539, SNB-19, SNB-75, and U251): IC_{50} = 0.023-0.069 μM<br>MGG8: IC_{50} = 0.014 μM<br>T98G: IC_{50} = 0.036 μM	- Inhibited tubulin polymerization (IC_{50} = 1.1 μM).<br>- Inhibited microtubule assembly in HCT-116 cells at 0.2 μM.<br>- Displayed IC_{50} values ranging from 0.047 to 0.024 μM against MGG8 cells and PD-glioma isolates that exhibit cancer stem cell properties.	- Reduced tumor growth by 200–300% in COLO205 xenograft model at 20 mg/kg.<br>- No toxicity towards COLO205 xenograft model at 20 mg/kg.<br>- Enhanced temozolide and radiation therapy effectiveness at 20 mg/kg.	- Plasma and brain concentration measurements of **34**, at 20 mg/kg in mice, revealed micromolar levels of **34**, a $T_{1/2}$ of approximately 6 h, and a brain/plasma ratio of 2.2, indicating blood-brain barrier penetraton.	[15]

**35 – 49**	**42, 44, 45, 48**:<br>MCF-7: EC_{50} = 0.007-0.0175 µM<br>**<u>MDR cancer cell lines</u>**:<br>MCF-7 TUBB3 βIII-tubulin: EC_{50} = 0.0026-0.0168 µM<br>**42-49**:<br>OVCAR-8: IC_{50} = 0.002-0.880 µM<br>**<u>MDR cancer cell lines</u>**:<br>NCI/ADR-RES: IC_{50} = 0.004-0.800 µM	**35-49:**<br>- Inhibited tubulin polymerization with IC_{50} values of 0.42-21 µM**.**<br>**42-45:**<br>- Inhibited tubulin polymerization at low micromolar concentrations (IC_{50} values of 0.42-0.49 µM).	**42**:<br>- Reduced tumor volume by 79% in a mouse xenograft model of MCF-7 TUBB3 at 25 mg/kg.<br>- No toxicity at 25 mg/kg in a mouse xenograft model of MCF-7 TUBB3 tumors.	ND	[16]
**50**	HCC-LM3: IC_{50} = 0.963 µM<br>RKO: IC_{50} = 0.17 µM<br>PANC-1: IC_{50} = 0.729 µM<br>SK-BR-3: IC_{50} = 0.431 µM<br>BT474: IC_{50} = 1.193 µM<br>SW620: IC_{50} = 0.673 µM<br>SW480: IC_{50} = 0.338 µM<br>SGC-7901: IC_{50} = 0.617 µM	- Inhibited tubulin polymerization at 10 µM.<br>- Caused cell shape to gradually turn from fusiform to round in RKO cells at 1 µM.<br>- Promoted G2/M cell cycle arrest in RKO, SW620, and PANC-1 cell lines (< 1 µM).<br>- Increased the percentage of total apoptotic cells in RKO and SW620 cell line at concentrations < 1 µM.<br>- Increased ROS levels 1.5 times in both RKO and SW620 cells at 0.5 µM.<br>- Disturbed HUVEC migration at 0.5 µM.	- Reduced tumor growth by 53.6% in SW620 xenograft mouse model at 25 mg/kg.<br>- No toxicity in SW620 xenograft mouse model at 25 mg/kg.	ND	[17]
**51 - 69**	HepG2: IC_{50} = 0.23-11.57 µM<br>HeLa: IC_{50} = 0.15-10.34 µM<br>MCF-7: IC_{50} = 0.38-13.13 µM<br>A549: IC_{50} = 0.3-8.55 µM<br>**<u>Non-cancer cell lines</u>**:<br>HEK-293T: IC_{50} = 126 µM	**51-69:**<br>- Inhibited tubulin polymerization (IC_{50} = 2.1-35.79 µM).<br>**57**:<br>- Induced G2/M arrested in HeLa cells (37.19%) at 0.1 µM.<br>- Decreased Cdc2, Cdc25c, and Cyclin B1 levels at 0.1 µM.<br>- Increased apoptosis rates to 14% in HeLa cells at 0.1 µM.<br>- Decreased the levels of anti-apoptotic proteins Bcl-2, Bcl-x1 and Mcl-1 at 0.1 µM.<br>- Low toxicity against non-cancer cell lines such as HEK 293T (IC_{50} of 126 µM).	ND	ND	[18]

**70**	**70**:<br>SGC-7901: IC_{50} = 0.09 μM<br>A549: IC_{50} = 0.65 μM<br>HeLa: IC_{50} = 0.268 μM	- Inhibited tubulin assembly at 2.5 μM.<br>- Caused destruction of microtubules and their wrapping around the nucleus in SGC-7901 cell lines at 0.18 μM.<br>- Caused G2/M cell cycle arrest in SGC-7901 cells at 0.18 μM.			[19]
**71, 72**	**71**:<br>A549: IC_{50} = 11.7 μM<br>MCF-7: IC_{50} = 14.85 μM<br>HepG2: IC_{50} = 63.43 μM<br>**<u>MDR cancer cell lines</u>**:<br>MCF-7 MX: IC_{50} = 14.94 μM<br>**72**:<br>A549: IC_{50} = 7.05 μM<br>MCF-7: IC_{50} = 9.88 μM<br>HepG2: IC_{50} = 21.97 μM<br>**<u>MDR cancer cell lines</u>**:<br>MCF-7 MX: IC_{50} = 20.2 μM	**71, 72**:<br>- Inhibited tubulin polymerization by 25% (**71**) and 42.85% (**72**), at 50 μM.<br>- Increased G2/M phase arrest to 29.78% (**71**) and 34.9% (**72**) in A549 cells at 10 μM.<br>**72**:<br>- Induced accumulation of total apoptotic cells (11.54%) in A549 treated cells at 10 μM.	ND	ND	[20]
**73-92**	**73-92:**<br>Panel of NCI-60 cell lines: GI_{50} <10 μM<br>**73**:<br>HeLa: IC_{50} = 3.2 μM	**73**:<br>- Induced G2/M phase arrest (54%) in Hela cells at 5 μM<br>- Induced microtubule depolymerization at 0.05 μM.<br>- Co-administration with an efflux pump inhibitor significantly increased intracellular retention.	ND	ND	[21]

**93, 94**	**93:**<br>MDA-MB-231: IC_{50} = 0.008 µM<br>MDA-MB-453: IC_{50} = 0.004 µM<br>SKBR3: IC_{50} = 0.0267 µM<br>A375: IC_{50} = 0.0059 µM<br>M14: IC_{50} = 0.0052 µM<br>RPMI7951: IC_{50} = 0.0052 µM<br>**94**:<br>MDA-MB-231: IC_{50} = 0.0022 µM<br>MDA-MB-453: IC_{50} = 0.0014 µM<br>SKBR3: IC_{50} = 0.0048 µM<br>A375: IC_{50} = 0.0018 µM<br>M14: IC_{50} = 0.0019 µM<br>RPMI7951: IC_{50} = 0.0024 µM<br>**MDR cancer cell lines**:<br>A375/TxR: IC_{50} = 0.0053 µM<br>MDA-MB-231/TxR: IC_{50} = 0.0021 µM<br>M14/LCC6MDR1: IC_{50} = 0.0049 µM	**94:**<br>- Inhibited tubulin polymerization at 10 µM.<br>**MDR cancer cell lines**:<br>- Maintained its antiproliferative activity against A375/TxR, MDA-MB-231/TxR and M14/LCC6MDR1 cell lines showing resistance indices of 1.04, 0.36 and 1.8, respectively.<br>- Reduced colony formation of A375/TxR cells, by 95% at 0.005 µM.<br>- Induced G2/M phase arrest (62%) in A375/TxR cells at 0.01 µM.<br>- Increased the proportion of apoptotic cells to 54.3% in A375/TxR cells at 0.01 µM.	**94:**<br>**MDR cancers:**<br>- Inhibited tumor growth by 44.4% in A375/TxR xenograft model at a dose of 10 mg/kg.<br>- Induced necrosis and reduced metastasis of tumors in A375/TxR xenograft model tumors.	**94:**<br>- Metabolic stability of 29.4 min in human liver microsomes.	[22]
**95, 96**	**95**:<br>MCF-7: EC_{50} = 1.5 µM<br>PANC-1: EC_{50} = 4.1 µM<br>**MDR cancer cell lines**<br>$NCI^{ADR/RES}$: EC_{50} = 1.5 µM<br>**96**:<br>NCI-60 Cell line EC_{50} < 2 µM<br>MCF-7: EC_{50} = 0.73 µM<br>PANC-1: EC_{50} = 1.1 µM<br>**MDR cancer cell lines:**	**95,96:**<br>- Inhibited tubulin polymerization.	ND	ND	[23]

	$NCI^{ADR/RES}$: EC_{50} = 0.81 μM				
**97**, **98**	**97**:<br>MDA-MB-231: IC_{50} = 3.2 μM<br>H1299: IC_{50} = 2.8 μM<br>PC-3: IC_{50} = 7 μM<br>HCC1806: IC_{50} = 8.4 μM<br>**Non-cancer cell lines**:<br>BJ tert: IC_{50} = 4 μM<br>**98**:<br>MDA-MB-231: IC_{50} = 4.8 μM<br>H1299: IC_{50} = 3.1 μM<br>PC-3: IC_{50} = 6.8 μM<br>HCC1806 = 7.2 μM<br>**Non-cancer cell lines**:<br>BJ tert: IC_{50} = 4.2 μM	**97, 98**:<br>- Inhibited tubulin polymerization.<br>- Interfered with the microtubule network in HCC1806 cells at 10 μM.<br>- Induced G_2/M phase arrest in HCC1806 cells (~50–60%) at 10 μM.<br>- Increased apoptosis and apoptotic markers in HCC1806, MDA-MB-231, H1299 and PC-3 cells at 10 μM.	**97, 98**:<br>- Reduced tumor size in HCC1806 breast cancer xenograft model at 2 mg/kg.	ND	[24]
**99**	MCF-7: IC_{50} = 3.4 μM<br>MDA-MB-23: IC_{50} = 12.2 μM<br>**Non-cancer cell lines:**<br>MCF-10A: IC_{50} = 23.6 μM	- Promoted microtubule disorganization in MCF-7 cells at 3.4 μM.<br>- Triggered apoptosis at 3.4 μM.<br>- Moderate toxicity against non-cancer cells.	ND	ND	[4]
**100**	A549: IC_{50} = 0.008 μM<br>HeLa: IC_{50} = 0.021 μM<br>MHCC-97H: IC_{50} = 0.006 μM<br>HCT-8: IC_{50} = 0.029 μM<br>A2780: IC_{50} = 0.018 μM<br>MCF-7: IC_{50} = 0.035 μM<br>**Non-cancer cell lines**:	- Inhibited tubulin polymerization (IC_{50} = 0.81 μM).<br>- Caused disturbance and shrinking of microtubules as well as condensation of chromatin and nucleus shrinkage in MHCC-97H cells at 0.005 μM.<br>- Promoted G2/M cell cycle arrest of MHCC-97H cells at 0.005 μM.<br>- Increased MHCC-97H apoptotic cells at 0.005 μM.<br>- Caused TrxR inhibition in MHCC-97H cells (IC_{50} = 10.24 μM) resulting in the accumulation of ROS and superoxide anions.	- Suppressed tumor growth by 75.4% in a cell line-derived xenograft mouse model at 30 mg/kg.	ND	[25]

	HUVECs: IC_{50} = 0.056 µM<br>LO2: IC_{50} = 0.398 µM<br>HLF: IC_{50} = 2.367 µM<br>MCF-10A: IC_{50} = 0.723 µM<br>**MDR cancer cell lines**:<br>MCF-7/ ADR: IC_{50} = 0.018 µM<br>HCT-8/VCR: IC_{50} = 0.045 µM<br>A2780/TAX: IC_{50} = 0.02 µM	- Downregulated the expression of anti-apoptotic proteins such as Bcl-2, and Bcl-xl in MHCC-97H cells at 0.01 µM.<br>- Upregulated the expression of pro-apoptotic proteins (Bax and Bad) in MHCC-97H cells at 0.01 µM.<br>- Showed low toxicity against human normal HUVECs, L-O2, HLF, and MCF-10A cells.			
**101, 102**	**101, 102:**<br>A549: IC_{50} = 0.094, 0.005 µM<br>KB: IC_{50} = 0.0527, 6.3 µM<br>K562: IC_{50} = 0.137, 7.8 µM<br>**MDR cancer cell lines**:<br>KB-VCR: IC_{50} = 0.0425, 0.0061 µM<br>KB-V1: IC_{50} = 0.0517, 0.0063 µM<br>K562-ADR: IC_{50} = 0.3052, 0.0071 µM<br>**102:**<br>HeLa: IC_{50} = 0.0042 µM<br>4T1: IC_{50} = 0.0077 µM<br>HT1080: IC_{50} = 0.0063 µM<br>RAW264.7: IC_{50} = 0.0076 µM<br>HL60: IC_{50} = 0.0068 µM<br>THP1: IC_{50} = 0.0074 µM<br>KB: IC_{50} = 0.0063 µM<br>**Non-cancer cell lines:**<br>MRC-9: IC_{50} = 0.1664 µM	**101, 102**:<br>- Inhibited tubulin polymerization by 23.9 % (**101**) and 75% (**102**) at 30 µM.<br>**102**:<br>- Disrupted microtubule network in A549 cells at 0.05 µM.<br>- Arrested more than 90% of cells of A549 cells in G_2/M phase at 0.02 µM.<br>- Selective towards cancer cells.	**102**:<br>- Inhibited tumor growth (T/C value 29.9%) in a murine 4T1 breast cancer xenograft model at 5 mg/kg.	**102**:<br>- Good metabolic stability, with a half-life of 60.3 h when incubated with human liver microsomes.	[26]

**103 - 107**	**103-107**:<br>MDA-MB-435: IC_{50} = 0.009-0.088 μM<br>HeLa: IC_{50} = 0.0135-0.146 μM<br>SK-OV-3: IC_{50} = 0.0118-0.136 μM<br>**MDR cancer cell lines**:<br>HeLa WTβ3: IC_{50} = 0.0106-0.108 μM<br>SK-OV-3 MDR1-M6/6: IC_{50} = 0.175-0.209 μM<br>**103**:<br>Panel of NCI-60 cell lines: IC_{50} = 0.0027 - 0.0175 μM	**103-107**:<br>- Inhibited tubulin assembly (IC_{50} = 0.49-2.3 μM).<br>- Rr values of ~1.0 in Hela and its βIII-tubulin expressing subline.<br>- Rr values (between 1-1.5) in SK-OV-3 ovarian carcinoma and its P-gp-expressing subline.	**103:**<br>- Antitumor effects in MDA-MB-435 xenograft model at 75 mg/kg.<br>- Moderate weight loss in MDA-MB-435 xenograft model at 75 mg/kg	ND	[27]
**108**	MCF-7: IC_{50} = 0.009 μM<br>HeLa: IC_{50} = 0.005 μM<br>Raji: IC_{50} = 0.013 μM<br>NCI-H460: IC_{50} = 0.014 μM<br>A2780S: IC_{50} = 0.008 μM<br>**MDR cancer cell lines**:<br>A2780T: IC_{50} = 0.0131 μM<br>A549T: IC_{50} = 0.0638 μM<br>MCF7-AD: IC_{50} = 0.0135 μM	- Did not induce α,β-tubulin degradation when HeLa cells were pretreated with 0.01 μM of a proteasome inhibitor (MG-132).<br>- Inhibited migration of HUVEC cells (76.21%) at 0.005 μM.<br>- Inhibited microtubule polymerization and spindle formation in A2780S cells at 0.01 μM.<br>**MDR cancer cell lines**:<br>- Induced G2/M phase arrest in A2780S (79.54%) and A2780T (72.89%) at 0.3 μM.<br>- Induced apoptosis in A2780S and A2780T cells at 0.3 μM.	- Inhibited tumor growth by 66.06% in A2780S xenograft mouse model at 10 mg/kg<br>**MDR cancers**:<br>- Inhibited tumor growth by 26.94% in A2780T xenograft mouse model at 10 mg/kg.<br>- IV administration showed no signs of toxicity at 10 mg/kg or 20 mg/kg.<br>- Oral administration showed signs of toxicity at 40mg/kg.	- Intravenous administration: $T_{1/2}$ = 3.57 hours<br>-Oral administration: $T_{1/2}$ = 4.42 hours<br>- Clearance rate for oral administration (5.06L/h/kg) higher than IV administration (1.52 L/h/kg).<br>- Oral bioavailability of 30.70 % in mice.	[28]
**109**	A549: IC_{50} = 0.01 μM<br>HeLa: IC_{50} = 0.02 μM<br>A2780: IC_{50} = 0.02 μM<br>HCT-8: IC_{50} = 0.04 μM<br>MCF-7: IC_{50} = 0.05 μM	- Inhibited tubulin polymerization (IC_{50} = 0.58 μM).<br>- Disrupted microtubule network in A549 cells at 0.005 μM.<br>- Induced G2/M phase arrest in A549 cells at 0.005 μM.<br>- Downregulated Cyclin B1 and consequently decreased Cdc2 and Cdc25C cells at 0.005 μM.	- Inhibited tumor growth by 78.63% (10 mg/kg) in A549 xenograft mouse models.	- Favorable oral (69.45%) bioavailability, fast absorption and a half-life of $T_{1/2}$ = 2.12 h for	[29]

	**Non-cancer cell lines**: HUVECs: IC_{50} = 0.04 μM LO2: IC_{50} = 1.45 μM HLF: IC_{50} = 1.32 μM MCF10-A: IC_{50} = 0.54 μM **MDR cancer cell lines**: A549/ADM: IC_{50} = 0.02 μM HCT-8/VCR: IC_{50} = 0.07 μM A2780/TAX: IC_{50} = 0.04 μM	- Triggered cell death in A549 cells mainly through cell ferroptosis at 0.005 μM. - Selective towards cancer cells.		PO, and $T_{1/2}$ = 0.62 h for IV.	
**110 - 113**	**110-113**: MDA-MB 231: IC_{50} = 0.43->10 μM HeLa: IC_{50} = 0.038-0.44 μM A549: IC_{50} = 0.043-0.76 μM HT-29: IC_{50} = 0.03-0.76 μM	**110 - 113**: - Inhibited tubulin polymerization (IC_{50} = 0.45-2.2 μM). **110**: - Promoted G2/M cell cycle arrest in HeLa cells at 0.01 μM. - Increased expression levels of Cyclin B and inhibited the phosphorylation of Cdc2 in HeLa cells at 0.01 μM. - Induced apoptosis in HeLa cells at 0.025 μM, with the appearance of cell necrosis at 0.050 μM (25% of cells became necrotic). - Decreased mitochondrial potential in HeLa cells at 0.025 μM.	**110**: - *In vivo*, zebrafish embryos showed no signs of toxicity after being exposed to 0.3 μM. - Decreased tumor cell dissemination and killed a significant portion of DiI-positive cancer cells.	ND	[30]
**114**	NCI-60 cell lines: GI_{50} ranging from > 100 to 0.0254 μM MCF-7: IC_{50} = 0.095 μM MDA-MB-231: IC_{50} = 0.62 μM **Non-cancer cell lines:** HEK-293T: IC_{50} > 50 μM	- Inhibited tubulin polymerization at 10 μM. - Selective towards cancer cells. - At 0.1 μM caused cell rounding, distinct abnormalities of the spindle formation as well as a loss of the structured tubulin in MCF-7 cells. - Induced cell apoptosis (14.2%) in MCF-7 cells at 0.1 μM. - Downregulated the expression of anti-apoptotic proteins Bcl2 and survivin in MCF-7 cells at 0.05 μM.	ND	ND	[31]

**115**	SW480: IC_{50} = 0.2 μM<br>CT26: IC_{50} = 0.43 μM<br>HT-29: IC_{50} = 0.28 μM<br>HCT-116: IC_{50} = 0.13 μM<br>HCT-15: IC_{50} = 0.31 μM<br>A549: IC_{50} = 0.41 μM<br>**<u>Non-cancer cell lines</u>:**<br>HUEVC: IC_{50} = 32.76 μM<br>L02: IC_{50} = 21.45 μM<br>**<u>MDR cancer cell lines</u>**:<br>A549/CDDP: IC_{50} = 0.85 μM<br>HCT-116/OXA: IC_{50} = 0.2 μM	- Inhibited tubulin polymerization (IC_{50} = 11.68 μM).- Inhibited HCT-116 cell migration (healing rate of 28.7% at 5 μM).<br>- Induced severe DNA damage in HCT-116/OXA cells at 5 μM.<br>- Promoted apoptosis in HCT116/OXA cells (apoptotic ratio of 51.9% at 5 μM).<br>- Increased intracellular ROS levels in HCT-116/OXA cells at 5 μM.<br>- Decreased mitochondrial membrane potential at 5 μM in HCT-116/OXA cells.<br>- Notable selectivity for cancer cell lines.	´ND	ND	[32]
**116**	HCT-116: IC_{50} = 0.023 μM<br>B16-F10: IC_{50} = 0.02 μM<br>HeLa: IC_{50} = 0.037 μM<br>HepG2: IC_{50} = 0.041 μM	- Inhibited tubulin polymerization (IC_{50} = 1.5 μM)<br>- B16-F10 cells after exposure to 0.025 μM displayed a lack of cellular structure and disrupted microtubule networks.<br>- Promoted G2/M cell cycle arrest in B16-F10 cells at 0.05 μM.<br>- Increased apoptotic cells in B16-F10 cells at 0.05 μM.<br>- Inhibit 100% of colony formation in BF16-F10 cells at 0.05 μM.	- Caused no significant weight loss at 10 mg/kg indicating a good safety profile in melanoma B16-F10 tumor model.<br>- B16-F10 tumor model treatment with a dose of 10 mg/kg caused tumor growth inhibition of 65.1 %, which was raised to 77.6% when combined with PD-L1 (10mg/kg) inhibitor.	ND	[33]
**117–128**	**117-128**<br>MDA-MB-435: IC_{50} = 0.00244-0.62 μM<br>HeLa: IC_{50} = 0.0279-2.6 μM<br>SK-OV-3: IC_{50} = 0.0342-3.3 μM	**117-128**:<br>Promoted microtubule depolymerization in A-10 cells (EC_{50} = 0.123-9.2 μM).<br>**<u>MDR cancer cell lines</u>**:<br>**125**:	**125**:<br>- Decreased tumor volume in an MDA-MB-435 xenograft mouse model at 50 mg/kg.	ND	[34]

	**<u>MDR cancer cell lines</u>**:<br>WTβIII: IC_{50} = 0.0338-2.6 μM<br>SKOV-3 MDR-1- M6/6: IC_{50} = 0.0392-3 μM	- Promoted microtubule depolymerization activity (EC_{50} = 0.123 μM) in A-10 cells.<br>- Overcame βIII tubulin-mediated drug resistance with a relative resistance value of 1.2 and was not affected by P-gp expression.			
**129-131**	**129-131:**<br>A375: IC_{50} = 0.001-0.0136 μM<br>M14: IC_{50} = 0.0006-0.0183 μM<br>MDA-MB-231: IC_{50} = 0.0018-0.0258 μM<br>MDA-MB-453: IC_{50} = 0.0006-0.0176 μM<br>Mia PaCa-2: IC_{50} = 0.0029-0.0444μM<br>PANC-1: IC_{50} = 0.0028-0.0562 μM<br>PC-3: IC_{50} = 0.0005-0.0239 μM<br>**<u>MDR cancer cell lines</u>**:<br>PC-3/TxR : IC_{50} = 0.0002-0.0144 μM	**131:**<br>- Inhibited tubulin polymerization at 5 and 10 μM.<br>- Caused G2/M arrest in PC-3 cells at 0.003 μM.<br>- Showed anti-angiogenic effect in HUVEC at 0.1 μM.<br>- Inhibited cell migration of PC-3 cells at 0.005 μM.<br>- Inhibited the growth of COS-7 cells without significant cytotoxicity. Cell viability of COS-7 cells after 3 μM treatment was above 50%.<br>- Selective towards cancer cells.	**131:**<br>**<u>MDR cancers</u>**:<br>- Reduced tumor volume by 85.6% in PC-3/TxR xenograft mouse model, while also inhibiting angiogenesis by 44% at a well-tolerated dose of 2.5 mg/kg.	**131**:<br>- Presented good stability and longer half-life than its parental compound ($T_{1/2}$ = >300 min) in liver microsomes.	[35]
**132**	MGC-803: IC_{50} = 0.0117 μM<br>HGC-27: IC_{50} = 0.0146 μM<br>SGC-7901: IC_{50} = 0.0211 μM<br>DU-145: IC_{50} = 0.1066 μM<br>PC-3: IC_{50} = 0.0299 μM<br>A549: IC_{50} = 0.0624 μM<br>NCI-H520: IC_{50} = 0.1107 μM<br>MCF-7: IC_{50} = 0.0525 μM<br>MDA-MB-231: IC_{50} = 0.3959 μM<br>EC-109: IC_{50} = 0.0289 μM<br>TE-1: IC_{50} = 0.2859 μM<br>HCT-116: IC_{50} = 0.0377 μM<br>SK-N-SH: IC_{50} = 0.0583 μM	- Induced G2/M arrest in MGC-803 and HGC-27 cell lines at 0.02 μM.<br>- Downregulated the expression of cell cycle-related proteins p-cdc2, Cyclin B1, CDC25B, Wee1 in MGC-803 and HGC-27 at 0.02 μM.<br>-Upregulated the expression of M−phase marker protein p-Histone H3 in MGC-803 and HGC-27 at 0.005 μM.<br>- Inhibited ERK1/2 proteins, RSK1, and TGF-β/SMAD signaling pathway in MGC-803 and HGC-27 cells at 0.02 μM.<br>- Increased apoptosis rate of MGC-803 and HGC-27 cells at 0.02 μM.<br>- Downregulated anti-apoptotic proteins (Bcl-2, Mcl-1, c-IAP1) expression in MGC-803 and HGC-27, at 0.005 μM.<br>Upregulated pro-apoptotic Noxa, and promoting cleavage of caspase-3/9 and PARP expression in MGC-803 and HGC-27 at 0.005 μM.	- Well tolerated at 20 mg/kg on mice bearing MGC-803 cells xenograft model, causing tumor growth inhibition of 85.9%, while downregulating the expression of c-myc, Ki67, and cyclinB1 and promoting PARP cleavage, indicating the induction of apoptosis.	ND	[36]

		- Reduced c-myc, Ki67, CyclinB1 expression and enhanced PARP cleavage in MGC-803 and HGC-27 at 0.005 μM.<br>- Inhibited MGC-803 and HGC-27 cell migration at 0.005 μM.<br>- Downregulated the expression levels of the cell migration-related proteins MMP2 and MMP9 at 0.003 μM.<br>- Decreased colony formation in MGC-803 and HGC-27 cell lines at 0.003 μM.			
**133**	MDA-MB-468: IC_{50} = 0.225 μM<br>MDA-MB-231: IC_{50} = 0.162 μM<br>MDA-MB-361: IC_{50} = 0.637 μM<br>MDA-MB-175-VII: IC_{50} = 0.43 μM<br>BT-549: IC_{50} = 0.182 μM<br>HCC70: IC_{50} = 0.352 μM<br>**Non-cancer cell lines:**<br>MCF10-A: IC_{50} = 4.409 μM<br>76N: IC_{50} = 4.156 μM	- Inhibited clonogenic viability in breast cancer cell lines (EC_{50} = 0.162-0.637 μM).<br>- More effective against breast cancer cells with mutant p53.<br>- Concentrations ≥ 1 μM caused G2/M phase arrest in both normal and cancer cells.<br>- Induced apoptosis in MDA-MB175-VII cells at 1 μM.<br>- Induced multi-micronuclei phenotype in MDA-MB-175-VII at 1 μM.<br>- Exhibited over 10-fold greater potency in breast cancer cells compared to normal mammary epithelial cells.	ND	ND	[37]
**134**, **135**	**134**:<br>A549: IC_{50} = 2.9 μM<br>MCF-7: IC_{50} = 7.17 μM<br>**Non-cancer cell lines:**<br>MRC-5: IC_{50} = 16.87 μM<br>**135**:<br>A549: IC_{50} = 2.33 μM<br>MCF-7: IC_{50} = 6.1 μM<br>**Non-cancer cell lines:**<br>MRC-5: IC_{50} = 12.1 μM	**134,135**:<br>- Demonstrated selectivity for cancer cells: selectivity indexes of 5.81 (**134**) and 5.20 (**135**).<br>- Stabilized tubulin polymerization at 20 μM.<br>- Stabilized microtubule polymerization in A549 cells at 20 μM.<br>- Induced in the G2/M phase arrest by 22% in A549 cells at 5 μM.<br>- Inhibited cancer cell migration in A549 cells at 2.5 μM.<br>- Inhibited the A549 cell colony formation at 5 μM.<br>- Increased apoptosis in A549 cells by 78.2% (**134**) and 91.4% (**135**) at 5 μM.<br>- Upregulated DeY-α-tubulin expression in A549 cancer cells.<br>- Downregulated Ac-α-tubulin expression in A549 cancer cells.	ND	**134,135**:<br>- *In silico* study showed agreement with the critical rules of drug-likeness and predicted poor oral bioavailability.	[38]

**136-138**	**<u>136-138</u>**<br>HeLa, EC, T24, SHSY-5Y, HepG2, HEC: dose dependent growth inhibitory effect at 0.1-10 μM	**136**:<br>- Restrained tubulin polymerization (IC_{50} = 16.12 μM).<br>- Disrupted the microtubule network of HepG2 cells at 1 μM.<br>- Induced G2/M phase arrest in HepG2 cells at 2 μM.<br>- Promoted apoptosis in HepG2 cells at 2 μM.	**136**:<br>- Tumor inhibitory ratios of 53.91% and 46.25% in H22 and S180 models, respectively, at 10 mg/kg.<br>- Low toxicity in H22 and S180 at 10/mg/kg.	ND	[39]
**139, 140**	**139:**<br>HT-29: IC_{50} = 2.2 μM<br>HepG2: IC_{50} = 4.64 μM<br>HeLa: IC_{50} = 2.84 μM<br>MCF-7: IC_{50} = 7.52 μM<br>**140:**<br>HT-29: IC_{50} = 17.52 μM<br>HepG2: IC_{50} = 11.51 μM<br>HeLa: IC_{50} = 10.9 μM<br>MCF-7: IC_{50} = 12.85 μM	**139, 140:**<br>- Disrupted microtubule morphology in HT-29 cells.<br>**139:**<br>- Inhibited tubulin polymerization.<br>- Induced G2/M phase arrest in HeLa cells at 4 μM.	ND	ND	[40]
**141**	A549: IC_{50} = 0.027 μM<br>Huh-7: IC_{50} = 0.03 μM<br>T24: IC_{50} = 0.046 μM<br>**<u>MDR cancer cell lines</u>**:<br>A549/Tax: IC_{50} = 0.033 μM	- Inhibited tubulin polymerization (IC_{50} = 1.75 μM).<br>- Prevented microtubule aggregation and spindle formation in A549 cells at 0.0125 μM.<br>- Induced G2/M phase accumulation in A549 cells at 0.0125 μM.<br>- Increased apoptosis in A549 cells at 0.0125 μM.<br>- Reduced A549 cell migration at 0.0125 μM.	- Reduced tumor volume by 64.1 % in A549 xenograft mouse models with no signs of toxicity after treatment with 25 mg/ kg.	ND	[41]
**142-147**	**142-147**<br>A549, KP-4, HeLa, BxPC-3 and MCF-7: IC_{50} = 0.009-1.8 μM<br>**143**:<br>A2780: IC_{50} = 0.075 μM	**142-146**:<br>- Inhibited tubulin polymerization with IC_{50} values of 4.2 (**142**), 1.3 (**143**), 8.8 (**144**), 1.6 (**145**), and 15.3 (**146**) μM.<br>- Strong inhibitory activity against JAK2.<br>**143**:	**143, 147**:<br>Reduced tumor weight by 70% (**143**) and 91% (**147**), in an A549 xenograft mouse model at 50 mg/kg.	**143**:<br>- *In Silico* testing showed favorable drug properties and stability.	[42]

	HCT-8: IC_{50} = 0.034 µM<br>MCF-7: IC_{50} = 0.018 µM<br>A549: IC_{50} = 0.08 µM<br>**MDR cancer cells**:<br>A2780/TAX: IC_{50} = 0.02 µM<br>HCT-8/VCR: IC_{50} = 0.049 µM<br>MCF-7/ADR: IC_{50} = 0.064 µM<br>A549/DDP: IC_{50} = 0.036 µM	- Induced G2/M cell cycle arrest in A549 cells at 0.005 (15.6%) and 0.02 (91.5%) µM.<br>- Selective towards cancer cells.<br>- Increased proportion of apoptotic A549 cells by 8.9% at 0.005 µM and 80.7% at 0.02 µM.<br>- Low resistance indices (0.3–0.36) when tested against drug-resistant cell lines (A2780/TAX, HCT-8/VCR, MCF-7/ADR, and A549/DDP) and their parental counterparts.<br>- Increased ROS production in A549 cells at 0.005 µM.<br>-Decreases mitochondrial membrane potential in A549 cells at 0.005 µM.		- Poor water solubility and limited oral bioavailability.<br>**147**:<br>Acceptable water solubility, oral bioavailability of 69.7%.	
**148**	HeLa: IC_{50} = 0.04 µM	- Disrupted tubulin polymerization at 4 µM.<br>- Induced G2/M phase arrest in HeLa cells at 0.01 µM.<br>- Inhibited Src signaling pathway, promoting a reduction in p-Src and p-FAK levels in HeLa cells at 0.05 µM.<br>- Demonstrated no significant cytotoxic effects and unaltered cell morphologies against MCF-10A, human fibroblast (CCD-18Co and BJ) cell lines at concentrations below 4 µM.	ND	- Showed excellent oral bioavailability in animal models (oral AUC value of 4.0 mg h/mL).<br>- Demonstrated good microsomal stability and a percentage of plasma protein binding within acceptable ranges in both mice and humans.	[43]
**149**	A549: IC_{50} = 3.12 µM<br>Caski: IC_{50} = 2.93 µM<br>MHCC-97H: IC_{50} = 1.09 µM<br>PC-3: IC_{50} = 3.52 µM<br>HCT-8: IC_{50} = 7.26 µM<br>**<u>MDR cancer cells</u>**:<br>MHCC-97H/CDDP: IC_{50} = 0.85 µM<br>PC-3/ENZR: IC_{50} = 4.22 µM<br>HCT-8/VCR: IC_{50} = 6.51 µM	- Inhibited tubulin polymerization (IC_{50} = 5.98 µM).<br>- Disrupted microtubule network causing the disappearance of filamentous structures, shrinkage, and punctate distribution in MHCC-97H cells at 0.75 µM.<br>- Increased G2/M arrest in MHCC-97H cells at 0.75 µM.<br>- Increased apoptosis in MHCC-97H cells at 0.75 µM.<br>- Elevated caspase-3 activity in MHCC-97H cells at 1.5 µM.<br>- Decreased mitochondrial membrane potential in MHCC-97H cells at 0.75 µM.<br>- Induced ROS accumulation in MHCC-97H at 0.75 µM.	ND	ND	[44]

	**Non-cancer cell lines**:<br>L-02: IC_{50} = 10.26 μM<br>RWPE-1: IC_{50} = 8.75 μM				
**150**	U87: IC_{50} = 0.9 μM<br>A549: IC_{50} = 0.88 μM<br>Huh7: IC_{50} = 1.19 μM	- Inhibited tubulin polymerization at 6 μM.<br>- Degraded the intracellular tubulin skeleton at 0.5 μM.<br>- Caused G2/M arrest in U87 (24.6%) at 1 μM.<br>- Increased percentage of apoptotic cells in U87 cells (11.28%) at 0.3125 μM.<br>- Downregulated TUBA1A and TUBA1B, as well as β-subunit related genes TUBB and TUBB4B.<br>- Inhibited U87 cell migration at 0.25 μM.	- Inhibited tumor growth and lowered tumor microvessel density (5 mg/kg) in mice bearing orthotropic glioma.<br>- No toxicity in mice bearing orthotropic glioma at doses up to 10 mg/kg.<br>-Showed positive correlation between blood-brain barrier permeability and *in vivo* anti-glioblastoma activity.	ND	[45]
**151, 152**	**151**:<br>HCT-15: IC_{50} = 0.0037 μM<br>NCI-H460: IC_{50} = 0.008 μM<br>MDA-MB-231: IC_{50} = 0.0104 μM<br>SUIT2: IC_{50} = 0.0154 μM<br>MIA PaCa-2: IC_{50} = 0.0099 μM<br>**152**:<br>HCT-15: IC_{50} = 0.0042 μM<br>NCI-H460: IC_{50} = 0.0083 μM<br>MDA-MB-231: IC_{50} = 0.0078 μM<br>SUIT2: IC_{50} = 0.0105 μM<br>MIA PaCa-2: IC_{50} = 0.0074 μM	**151, 152**:<br>- Induced cell cycle arrest in MIA PaCa-2 PDAC cells at 0.025 μM.<br>- Retained nanomolar-range efficacy against hM1A patient-derived PDAC organoid line.	ND	ND	[46]

**153**	HeLa: IC_{50} = 0.05 μM<br>HCT 116: IC_{50} = 0.03 μM<br>MCF-7: IC_{50} = 0.02 μM<br>K562: IC_{50} = 0.03 μM<br>Molm 13: IC_{50} = 0.02 μM<br>A2780S: IC_{50} = 0.018 μM<br>A549 IC_{50} = 0.034 μM<br>**<u>MDR cancer cell lines</u>**:<br>A2780T: IC_{50} = 0.016 μM<br>A549T: IC_{50} = 0.037 μM<br>MCF-7/ADR: IC_{50} = 0.032 μM	- Caused tubulin degradation in HeLa cells at 1 μM.<br>- Induced pro-apoptotic effects in A2780S cells at 0.3 μM.<br>- Induced G2/M phase arrest in A2780S at 0.1 μM.<br>**<u>MDR cancer cell lines</u>**:<br>-Maintained antiproliferative potency against multidrug-resistant cell lines.<br>- Induced G2/M phase arrest in A2780T cell lines at 0.1 μM.	- Inhibited tumor growth by 45.3% in A2780S xenograft mouse model at 10 mg/kg.<br>**<u>MDR cancers</u>:**<br>- Inhibited tumor growth by 52.2% in A2780T xenograft mouse model at 10 mg/kg.	- $T_{1/2}$ values of 1.93, 5.94 h, and Cmax values of 2885.77 and 77.92 μg/L for intravenous and oral administration, respectively, in rats at 10 mg/kg.<br>- Bioavailability values for oral administration reached 10.47% in rats.	[47]
**154-157**	**154:**<br>MDA-MB-231: IC_{50} = 30.4 μM<br>**155:**<br>MDA-MB-231: IC_{50} = 18.98 μM<br>**156:**<br>MDA-MB-231: IC_{50} = 34.97 μM<br>**157:**<br>MDA-MB-231: IC_{50} = 14.54 μM	ND	ND	- Favorable physicochemical features and pharmacokinetic profile in *in silico* studies.	[48]
**158-167**	**158-167:**<br>K562: IC_{50} = 0.53-74.48 μM<br>HCT-116: IC_{50} = 19.54-86.36 μM<br>HL-60: IC_{50} = 0.35-37.91 μM<br>H1299: IC_{50} = 0.3-18.03 μM<br>**161:**<br>K562: IC_{50} = 0.53 μM<br>HCT-116: IC_{50} = 24.61 μM<br>HL-60: IC_{50} = 0.35 μM	ND	ND	ND	[49]

	H1299: IC_{50} < 0.3 μM				
**168**	Panel of breast, ovarian, lung, stomach, cervical, pancreatic, prostate, colon and malignant glioma cancer cell lines: IC_{50} = 0.0005-0.015 μM<br>MCF-7: IC_{50} = 0.0014 μM<br>**<u>MDR cancer cell lines</u>**:<br>A2780/T: IC_{50} = 0.0048 μM<br>A549/T: IC_{50} = 0.0042 μM<br>A2780/CDDP: IC_{50} = 0.0016 μM<br>A549/CDDP: IC_{50} = 0.0020 μM	- Hindered tubulin polymerization (IC_{50} = 2 μM).<br>- Disorganized fibrous microtubule structures and decreased their density in MCF-7 cells at 0.001 μM.<br>- Induced G2/M phase arrest in MCF-7 cells (67%) after treatment with 0.01 μM.<br>- Increased cyclin-B1, CDK-1, and P-Histone H3 proteins at 0.01 μM.<br>- Increased apoptosis rates in MCF-7 cells (46%) at 0.01 μM.<br>- Decreased colony formation of MCF-7 cells after at 0.005 μM.<br>- Delayed cell migration of MDA-MB-231 cells and downregulated the expression of the metastasis-related proteins vimentin and MMP-9 at 0.01 μM.<br>**<u>MDR cancer cell lines</u>**:<br>- Maintained antiproliferative potency in multidrug resistant cell lines A2780/T, A549/T, A2780/CDDP and A549/CDDP with resistance indexes between 1.35-1.81.	- Reduced tumor weight by 63% in MDA-MB-231 xenograft mice model at 7.5 mg/kg.<br>- Showed no significant signs of toxicity in MDA-MB-231 xenograft mice model at doses up to 15 mg/kg.<br>- Caused development of necrosis in tumors.<br>**<u>MDR cancers</u>**:<br>- Reduced tumor weight by 74% in A549/T xenograft mode with no reduction in body weight at a dose of 15 mg/kg.	- Area under the concentration−time curve of 1335 ng/mL, $T_{1/2}$=1.92, maximum plasma concentration of 1196 ng/mL in mice.<br>- Oral bioavailability of approximately 40% in mice.	[50]
**169**	NCI-60 cancer cell line panel: average GI_{50} values of 0.0219 μM<br>HT-29: IC_{50} = 0.004 μM<br>**<u>Non-cancer cell lines</u>:**<br>PBMCs: IC_{50} = 14.064 μM<br>MRC-5: IC_{50} = 23.256 μM	- Showed selectivity for cancer cell lines.<br>- Induced disassembly of the microtubule network in A549 cells at 0.25 μM.<br>- Induced G2/M cell cycle arrest in Jurkat T-cell leukemia cells at 0.002 μM.<br>- Induced apoptosis in Jurkat T-cell leukemia cells at 0.01 μM	- Inhibited tumor growth in a HT-29 mouse xenograft model with a T/C ratio of 42% at 15 mg/kg.<br>- Showed no significant signs of toxicity in a HT-29 mouse xenograft model at doses up to 30 mg/kg.		[51]
**170**	A549: IC_{50} = 0.023 μM<br>**<u>MDR cancer cell lines</u>:**<br>A549/T: IC_{50} = 0.057 μM	- Displayed a resistance index of 2.47 against A549/T its parental subline.<br>- Inhibited tubulin polymerization (IC_{50} = 1.8 μM).	- Suppressed tumor growth in A549/T xenograft mouse		[52]

	**<u>Non-cancer cell lines</u>**:<br>MCF-10A cells: inhibitory rate of 20% at 0.01 μM.	- Disrupted microtubule structures in A549 and A549/T cells at 0.05 and 0.1 μM, respectively.<br>- Induced G2/M cell cycle arrest in A549 and A549/T at 0.025 μM.<br>- Promoted apoptosis in A549 (15.47% at 0.025 μM) and A549/T (22.2% at 0.0625 μM) cells.	model at 15 mg/kg and 30 mg/kg.		
MTAs targeting maytansine binding site					
**171-180**	**171-178:**<br>A549: IC_{50} = 0.00007->1.030 μM<br>A2780: IC_{50} = 0.000033-2.088 μM<br>**<u>MDR cancer cell lines</u>**:<br>A2780AD: IC_{50} between 0.0047->19.706 μM<br>**173**:<br>A549: IC_{50} = 0.00024 μM<br>A2780: IC_{50} = 0.00011 μM<br>**<u>MDR cancer cell lines</u>**:<br>A2780AD: IC_{50} = 0.011 μM	**171-178:**<br>- Inhibited tubulin assembly at 27.5 μM.<br>**173:**<br>- At 0.01 μM, disrupted microtubule networks in A549 cells, originating signs of depolymerization during interphase in irregular bi-nucleated cells.	ND	ND	[53]
MTAs targeting laulimalide / peloruside A binding site					
**181**	HeLa: 36.7% of growth inhibition at 2 μM, HL-60: 59.67 % of growth inhibition at 2.5 μM	- Promoted tubulin polymerization at 0.025 μM.<br>- Increased the density of microtubule networks and initiated bundling of microtubules in HeLa and HL-60 cells at 0.8 μM and 2.5 μM, respectively.<br>- Increased of oblate and lobulated nuclei in HeLa cells and of lobulated nuclei in HL60-cells at 0.8 μM and 2.5 μM, respectively.<br>- Promoted G2/M cell cycle arrest in HeLa (14%) and HL-60 (37.4%) cells at 1.5 μM.	ND	ND	[54]

CA-4: Combretastatin A4; MDR: multidrug resistance; ND: Not disclosed; ROS: Reactive oxygen species; T/C: Treated over control; Rr: Relative resistance; IV: Intravenous; DNA: Deoxyribonucleic acid; AUC: Area under curve; PO: Oral administration

References

1. Rong, D.; Wang, C.; Zhang, X.; Wei, Y.; Zhang, M.; Liu, D.; Farhan, H.; Momen Ali, S.A.; Liu, Y.; Taouil, A.; Guo, W.; Wang, Y.; Ojima, I.; Yang, S.; Wang, H. A novel taxane, difluorovinyl-ortataxel, effectively overcomes paclitaxel-resistance in breast cancer cells. *Cancer Lett* **2020**, *491*, 36-49, doi:10.1016/j.canlet.2020.06.025.
2. Zhou, X.; Fu, Y.H.; Zou, Y.Y.; Meng, J.; Ou-Yang, G.P.; Ge, Q.S.; Wang, Z.C. Discovery of Simple Diacylhydrazine-Functionalized Cinnamic Acid Derivatives as Potential Microtubule Stabilizers. *Int J Mol Sci* **2022**, *23*, doi:10.3390/ijms232012365.
3. Singh, A.; Chang, T.Y.; Kaur, N.; Hsu, K.C.; Yen, Y.; Lin, T.E.; Lai, M.J.; Lee, S.B.; Liou, J.P. CAP rigidification of MS-275 and chidamide leads to enhanced antiproliferative effects mediated through HDAC1, 2 and tubulin polymerization inhibition. *Eur J Med Chem* **2021**, *215*, 113169, doi:10.1016/j.ejmech.2021.113169.
4. Sinicropi, M.S.; Tavani, C.; Rosano, C.; Ceramella, J.; Iacopetta, D.; Barbarossa, A.; Bianchi, L.; Benzi, A.; Maccagno, M.; Ponassi, M.; Spinelli, D.; Petrillo, G. A Nitrocarbazole as a New Microtubule-Targeting Agent in Breast Cancer Treatment. *Applied Sciences* **2021**, *11*, doi:10.3390/app11199139.
5. Cui, Y.J.; Ma, C.C.; Zhang, C.M.; Tang, L.Q.; Liu, Z.P. The discovery of novel indazole derivatives as tubulin colchicine site binding agents that displayed potent antitumor activity both in vitro and in vivo. *Eur J Med Chem* **2020**, *187*, 111968, doi:10.1016/j.ejmech.2019.111968.
6. Malebari, A.M.; Fayne, D.; Nathwani, S.M.; O'Connell, F.; Noorani, S.; Twamley, B.; O'Boyle, N.M.; O'Sullivan, J.; Zisterer, D.M.; Meegan, M.J. beta-Lactams with antiproliferative and antiapoptotic activity in breast and chemoresistant colon cancer cells. *Eur J Med Chem* **2020**, *189*, 112050, doi:10.1016/j.ejmech.2020.112050.
7. Jian, X.E.; Yang, F.; Jiang, C.S.; You, W.W.; Zhao, P.L. Synthesis and biological evaluation of novel pyrazolo[3,4-b]pyridines as cis-restricted combretastatin A-4 analogues. *Bioorg Med Chem Lett* **2020**, *30*, 127025, doi:10.1016/j.bmcl.2020.127025.
8. Lin, B.Y.; Liu, W.L.; Huang, H.; Hu, Y.G.; Gong, S.; Meng, Y.H.; Yan, J.; Lu, Y.Z.; Chen, H.L. AQ-4, a deuterium-containing molecule, acts as a microtubule-targeting agent for cancer treatment. *Eur J Pharmacol* **2020**, *877*, 173093, doi:10.1016/j.ejphar.2020.173093.
9. Han, H.J.; Park, C.; Hwang, J.; N, R.T.; Kim, S.O.; Han, J.; Woo, M.; B, S.; Ryoo, I.J.; Lee, K.H.; Cha-Molstad, H.; Kwon, Y.T.; Kim, B.Y.; Soung, N.K. CPPF, A Novel Microtubule Targeting Anticancer Agent, Inhibits the Growth of a Wide Variety of Cancers. *Int J Mol Sci* **2020**, *21*, doi:10.3390/ijms21134800.
10. He, J.; Zhang, M.; Tang, L.; Liu, J.; Zhong, J.; Wang, W.; Xu, J.P.; Wang, H.T.; Li, X.F.; Zhou, Z.Z. Synthesis, Biological Evaluation, and Molecular Docking of Arylpyridines as Antiproliferative Agent Targeting Tubulin. *ACS Med Chem Lett* **2020**, *11*, 1611-1619, doi:10.1021/acsmedchemlett.0c00278.
11. Du, T.; Lin, S.; Ji, M.; Xue, N.; Liu, Y.; Zhang, Z.; Zhang, K.; Zhang, J.; Zhang, Y.; Wang, Q.; Sheng, L.; Li, Y.; Lu, D.; Chen, X.; Xu, H. A novel orally active microtubule destabilizing agent S-40 targets the colchicine-binding site and shows potent antitumor activity. *Cancer Lett* **2020**, *495*, 22-32, doi:10.1016/j.canlet.2020.08.040.

12. Kode, J.; Kovvuri, J.; Nagaraju, B.; Jadhav, S.; Barkume, M.; Sen, S.; Kasinathan, N.K.; Chaudhari, P.; Mohanty, B.S.; Gour, J.; Sigalapalli, D.K.; Ganesh Kumar, C.; Pradhan, T.; Banerjee, M.; Kamal, A. Synthesis, biological evaluation, and molecular docking analysis of phenstatin based indole linked chalcones as anticancer agents and tubulin polymerization inhibitors. *Bioorg Chem* **2020**, *105*, 104447, doi:10.1016/j.bioorg.2020.104447.
13. Ibrahim, T.S.; Hawwas, M.M.; Malebari, A.M.; Taher, E.S.; Omar, A.M.; O'Boyle, N.M.; McLoughlin, E.; Abdel-Samii, Z.K.; Elshaier, Y. Potent Quinoline-Containing Combretastatin A-4 Analogues: Design, Synthesis, Antiproliferative, and Anti-Tubulin Activity. *Pharmaceuticals (Basel)* **2020**, *13*, doi:10.3390/ph13110393.
14. Choudhary, S.; Doshi, A.; Luckett-Chastain, L.; Ihnat, M.; Hamel, E.; Mooberry, S.L.; Gangjee, A. Potential of substituted quinazolines to interact with multiple targets in the treatment of cancer. *Bioorg Med Chem* **2021**, *35*, 116061, doi:10.1016/j.bmc.2021.116061.
15. Horne, E.A.; Diaz, P.; Cimino, P.J.; Jung, E.; Xu, C.; Hamel, E.; Wagenbach, M.; Kumasaka, D.; Wageling, N.B.; Azorin, D.D.; Winkler, F.; Wordeman, L.G.; Holland, E.C.; Stella, N. A brain-penetrant microtubule-targeting agent that disrupts hallmarks of glioma tumorigenesis. *Neurooncol Adv* **2021**, *3*, vdaa165, doi:10.1093/noajnl/vdaa165.
16. Islam, F.; Quadery, T.M.; Bai, R.; Luckett-Chastain, L.R.; Hamel, E.; Ihnat, M.A.; Gangjee, A. Novel pyrazolo[4,3-d]pyrimidine microtubule targeting agents (MTAs): Synthesis, structure-activity relationship, in vitro and in vivo evaluation as antitumor agents. *Bioorg Med Chem Lett* **2021**, *41*, 127923, doi:10.1016/j.bmcl.2021.127923.
17. Zhu, H.; Ying, S.; Zhou, B.; Liang, X.; He, Q.; Song, P.; Hu, X.; Shi, K.; Xiong, M.; Jin, H.; Pan, Y. Discovery of novel 2-aryl-3-sulfonamido-pyridines (HoAns) as microtubule polymerization inhibitors with potent antitumor activities. *Eur J Med Chem* **2021**, *211*, 113117, doi:10.1016/j.ejmech.2020.113117.
18. Wu, M.K.; Man, R.J.; Liao, Y.J.; Zhu, H.L.; Zhou, Z.G. Discovery of novel indole-1,2,4-triazole derivatives as tubulin polymerization inhibitors. *Drug Dev Res* **2021**, *82*, 1008-1020, doi:10.1002/ddr.21805.
19. Wang, C.; Li, Y.; Liu, Z.; Wang, Z.; Liu, Z.; Man, S.; Zhang, Y.; Bao, K.; Wu, Y.; Guan, Q.; Zuo, D.; Zhang, W. Design, synthesis and biological evaluation of 1-Aryl-5-(4-arylpiperazine-1-carbonyl)-1H-tetrazols as novel microtubule destabilizers. *J Enzyme Inhib Med Chem* **2021**, *36*, 549-560, doi:10.1080/14756366.2020.1759582.
20. Rahimzadeh Oskuei, S.; Mirzaei, S.; Reza Jafari-Nik, M.; Hadizadeh, F.; Eisvand, F.; Mosaffa, F.; Ghodsi, R. Design, synthesis and biological evaluation of novel imidazole-chalcone derivatives as potential anticancer agents and tubulin polymerization inhibitors. *Bioorg Chem* **2021**, *112*, 104904, doi:10.1016/j.bioorg.2021.104904.
21. Riu, F.; Sanna, L.; Ibba, R.; Piras, S.; Bordoni, V.; Scorciapino, M.A.; Lai, M.; Sestito, S.; Bagella, L.; Carta, A. A comprehensive assessment of a new series of 5',6'-difluorobenzotriazole-acrylonitrile derivatives as microtubule targeting agents (MTAs). *Eur J Med Chem* **2021**, *222*, 113590, doi:10.1016/j.ejmech.2021.113590.
22. Chen, H.; Deng, S.; Albadari, N.; Yun, M.K.; Zhang, S.; Li, Y.; Ma, D.; Parke, D.N.; Yang, L.; Seagroves, T.N.; White, S.W.; Miller, D.D.; Li, W. Design, Synthesis, and Biological Evaluation of Stable Colchicine-Binding Site Tubulin Inhibitors 6-Aryl-2-benzoyl-pyridines as Potential Anticancer Agents. *J Med Chem* **2021**, *64*, 12049-12074, doi:10.1021/acs.jmedchem.1c00715.
23. Yong, C.; Devine, S.M.; Abel, A.C.; Tomlins, S.D.; Muthiah, D.; Gao, X.; Callaghan, R.; Steinmetz, M.O.; Prota, A.E.; Capuano, B.; Scammells, P.J. 1,3-Benzodioxole-Modified Noscapine Analogues: Synthesis, Antiproliferative Activity, and Tubulin-Bound Structure. *ChemMedChem* **2021**, *16*, 2882-2894, doi:10.1002/cmdc.202100363.

24. Boichuk, S.; Galembikova, A.; Syuzov, K.; Dunaev, P.; Bikinieva, F.; Aukhadieva, A.; Zykova, S.; Igidov, N.; Gankova, K.; Novikova, M.; Kopnin, P. The Design, Synthesis, and Biological Activities of Pyrrole-Based Carboxamides: The Novel Tubulin Inhibitors Targeting the Colchicine-Binding Site. *Molecules* **2021**, *26*, doi:10.3390/molecules26195780.
25. Yan, J.; Xu, Y.; Jin, X.; Zhang, Q.; Ouyang, F.; Han, L.; Zhan, M.; Li, X.; Liang, B.; Huang, X. Structure modification and biological evaluation of indole-chalcone derivatives as anti-tumor agents through dual targeting tubulin and TrxR. *Eur J Med Chem* **2022**, *227*, 113897, doi:10.1016/j.ejmech.2021.113897.
26. Cheng, B.; Zhu, G.; Meng, L.; Wu, G.; Chen, Q.; Ma, S. Identification and optimization of biphenyl derivatives as novel tubulin inhibitors targeting colchicine-binding site overcoming multidrug resistance. *Eur J Med Chem* **2022**, *228*, 113930, doi:10.1016/j.ejmech.2021.113930.
27. Islam, F.; Doshi, A.; Robles, A.J.; Quadery, T.M.; Zhang, X.; Zhou, X.; Hamel, E.; Mooberry, S.L.; Gangjee, A. Design, Synthesis, and Biological Evaluation of 5,6,7,8-Tetrahydrobenzo[4,5]thieno[2,3-d]pyrimidines as Microtubule Targeting Agents. *Molecules* **2022**, *27*, doi:10.3390/molecules27010321.
28. Li, Y.; Liu, Y.; Zhu, Z.; Yan, W.; Zhang, C.; Yang, Z.; Bai, P.; Tang, M.; Shi, M.; He, W.; Fu, S.; Liu, J.; Han, K.; Li, J.; Xie, L.; Ye, H.; Yang, J.; Chen, L. Structure-Based Design and Synthesis of N-Substituted 3-Amino-beta-Carboline Derivatives as Potent alphabeta-Tubulin Degradation Agents. *J Med Chem* **2022**, *65*, 2675-2693, doi:10.1021/acs.jmedchem.1c02159.
29. Zhou, J.; Pang, Y.; Zhang, W.; OuYang, F.; Lin, H.; Li, X.; Yan, J. Discovery of a Novel Stilbene Derivative as a Microtubule Targeting Agent Capable of Inducing Cell Ferroptosis. *J Med Chem* **2022**, *65*, 4687-4708, doi:10.1021/acs.jmedchem.1c01775.
30. Romagnoli, R.; Oliva, P.; Prencipe, F.; Manfredini, S.; Budassi, F.; Brancale, A.; Ferla, S.; Hamel, E.; Corallo, D.; Aveic, S.; Manfreda, L.; Mariotto, E.; Bortolozzi, R.; Viola, G. Design, Synthesis and Biological Investigation of 2-Anilino Triazolopyrimidines as Tubulin Polymerization Inhibitors with Anticancer Activities. *Pharmaceuticals (Basel)* **2022**, *15*, doi:10.3390/ph15081031.
31. Malebari, A.M.; Duffy Morales, G.; Twamley, B.; Fayne, D.; Khan, M.F.; McLoughlin, E.C.; O'Boyle, N.M.; Zisterer, D.M.; Meegan, M.J. Synthesis, Characterisation and Mechanism of Action of Anticancer 3-Fluoroazetidin-2-ones. *Pharmaceuticals* **2022**, *15*, 1044, doi:10.3390/ph15091044.
32. Cao, X.; Li, R.; Wang, H.; Guo, C.; Wang, S.; Chen, X.; Zhao, R. Novel indole–chalcone platinum(IV) complexes as tubulin polymerization inhibitors to overcome oxaliplatin resistance in colorectal cancer. *Journal of Molecular Structure* **2023**, *1272*, doi:10.1016/j.molstruc.2022.134169.
33. Peng, X.; Ren, Y.; Pan, W.; Liu, J.; Chen, J. Discovery of Novel Acridane-Based Tubulin Polymerization Inhibitors with Anticancer and Potential Immunomodulatory Effects. *J Med Chem* **2023**, *66*, 627-640, doi:10.1021/acs.jmedchem.2c01566.
34. Choudhary, S.; Kaku, K.; Robles, A.J.; Hamel, E.; Mooberry, S.L.; Gangjee, A. Simple monocyclic pyrimidine analogs as microtubule targeting agents binding to the colchicine site. *Bioorg Med Chem* **2023**, *82*, 117217, doi:10.1016/j.bmc.2023.117217.
35. Pochampally, S.; Hartman, K.L.; Wang, R.; Wang, J.; Yun, M.K.; Parmar, K.; Park, H.; Meibohm, B.; White, S.W.; Li, W.; Miller, D.D. Design, Synthesis, and Biological Evaluation of Pyrimidine Dihydroquinoxalinone Derivatives as Tubulin Colchicine Site-Binding Agents That Displayed Potent Anticancer Activity Both In Vitro and In Vivo. *ACS Pharmacol Transl Sci* **2023**, *6*, 526-545, doi:10.1021/acsptsci.2c00108.

36. Song, J.; Wang, S.Y.; Wang, X.; Jia, M.Q.; Tian, X.Y.; Fu, X.J.; Jin, C.Y.; Zhang, S.Y. Discovery of a novel Coumarin-Dihydroquinoxalone derivative MY-673 as a tubulin polymerization inhibitor capable of inhibiting the ERK pathway with potent anti-gastric cancer activities. *Bioorg Chem* **2023**, *137*, 106580, doi:10.1016/j.bioorg.2023.106580.
37. Graff, B.T.; Palanivel, C.; Jenkins, C.B.; Baranowska-Kortylewicz, J.; Yan, Y. Benzimidazole carbamate induces cytotoxicity in breast cancer cells via two distinct cell death mechanisms. *Cell Death Discov* **2023**, *9*, 162, doi:10.1038/s41420-023-01454-6.
38. Song, I.H.; Park, S.J.; Yeom, G.S.; Song, K.S.; Kim, T.; Nimse, S.B. Not all benzimidazole derivatives are microtubule destabilizing agents. *Biomed Pharmacother* **2023**, *164*, 114977, doi:10.1016/j.biopha.2023.114977.
39. Song, J.; Liu, S.; Ren, Y.; Zhang, X.; Zhao, B.; Wang, X.; Li, Y. Organotin benzohydroxamate derivatives (OTBH) target colchicine-binding site exerting potent antitumor activity both in vitro and vivo revealed by quantitative proteomic analysis. *Eur J Pharm Sci* **2023**, *187*, 106488, doi:10.1016/j.ejps.2023.106488.
40. Dong, H.; Lu, L.; Song, X.; Li, Y.; Zhou, J.; Xu, Y.; Zhang, Y.; Qi, J.; Liang, T.; Wang, J. Design, synthesis and biological evaluation of tetrahydroquinoxaline sulfonamide derivatives as colchicine binding site inhibitors. *RSC Adv* **2023**, *13*, 30202-30216, doi:10.1039/d3ra05720h.
41. Cui, Y.J.; Zhou, Y.; Zhang, X.W.; Dou, B.K.; Ma, C.C.; Zhang, J. The discovery of water-soluble indazole derivatives as potent microtubule polymerization inhibitors. *Eur J Med Chem* **2023**, *262*, 115870, doi:10.1016/j.ejmech.2023.115870.
42. Chen, L.; Hu, Y.; Lu, Z.; Lin, Z.; Li, L.; Wu, J.Q.; Yu, Z.L.; Wang, C.; Chen, W.H.; Hu, J. Design, Synthesis, and Antitumor Efficacy of Substituted 2-Amino[1,2,4]triazolopyrimidines and Related Heterocycles as Dual Inhibitors for Microtubule Polymerization and Janus Kinase 2. *J Med Chem* **2023**, *66*, 15006-15024, doi:10.1021/acs.jmedchem.3c01690.
43. Park, J.; Kang, M.; Lim, A.; Cho, K.J.; Chae, C.H.; Koh, B.; Jeon, H. Synthesis and evaluation of tirbanibulin derivatives: a detailed exploration of the structure-activity relationship for anticancer activity. *RSC Adv* **2023**, *13*, 35583-35591, doi:10.1039/d3ra06790d.
44. Hu, S.; Li, Y.; Zhou, J.; Xu, K.; Pang, Y.; Weiskirchen, R.; Ocker, M.; Ouyang, F. Identification of acetylshikonin as a novel tubulin polymerization inhibitor with antitumor activity in human hepatocellular carcinoma cells. *J Gastrointest Oncol* **2023**, *14*, 2574-2586, doi:10.21037/jgo-23-842.
45. Yang, H.; Zhang, D.; Yuan, Z.; Qiao, H.; Xia, Z.; Cao, F.; Lu, Y.; Jiang, F. Novel 4-Aryl-4H-chromene derivative displayed excellent in vivo anti-glioblastoma efficacy as the microtubule-targeting agent. *Eur J Med Chem* **2024**, *267*, 116205, doi:10.1016/j.ejmech.2024.116205.
46. Homer, J.A.; Koelln, R.A.; Barrow, A.S.; Gialelis, T.L.; Boiarska, Z.; Steinohrt, N.S.; Lee, E.F.; Yang, W.H.; Johnson, R.M.; Chung, T.; Habowski, A.N.; Vishwakarma, D.S.; Bhunia, D.; Avanzi, C.; Moorhouse, A.D.; Jackson, M.; Tuveson, D.A.; Lyons, S.K.; Lukey, M.J.; Fairlie, W.D.; Haider, S.M.; Steinmetz, M.O.; Prota, A.E.; Moses, J.E. Modular synthesis of functional libraries by accelerated SuFEx click chemistry. *Chem Sci* **2024**, *15*, 3879-3892, doi:10.1039/d3sc05729a.
47. Zhang, C.; Yan, W.; Liu, Y.; Tang, M.; Teng, Y.; Wang, F.; Hu, X.; Zhao, M.; Yang, J.; Li, Y. Structure-based design and synthesis of BML284 derivatives: A novel class of colchicine-site noncovalent tubulin degradation agents. *Eur J Med Chem* **2024**, *268*, 116265, doi:10.1016/j.ejmech.2024.116265.

48. Doan, N.Q.H.; Tran, H.N.; Nguyen, N.T.M.; Nguyen, K.D.T.; Tao, V.M.; Lai, N.N.; Tran, H.T.T.; Luu, P.H.T. Design, synthesis, and evaluation of anti-breast cancer activity of colchicine - combretastatin A-4 analogues containing quinoline as microtubule-targeting agents. *Journal of Molecular Structure* **2024**, *1312*, 138465, doi:10.1016/j.molstruc.2024.138465.
49. Silva, W.P.d.; Caiana, R.R.A.; Barros, M.E.S.B.; Freitas, J.C.R.; da Silva, P.B.N.; Militão, G.G.C.; Oliveira, R.A.; Menezes, P.H. Design, stereoselective synthesis, and antitumoral activity of combretastatin A-4 analogs. *Results in Chemistry* **2024**, *7*, doi:10.1016/j.rechem.2024.101539.
50. Jiang, F.; Yu, M.; Liang, Y.; Ding, K.; Wang, Y. Discovery of Novel Diaryl-Substituted Fused Heterocycles Targeting Katanin and Tubulin with Potent Antitumor and Antimultidrug Resistance Efficacy. *J Med Chem* **2024**, *67*, 12118-12142, doi:10.1021/acs.jmedchem.4c00878.
51. Herman, J.; Vanstreels, E.; Bardiot, D.; Prota, A.E.; Gaillard, N.; Gao, L.J.; Vercruysse, T.; Persoons, L.; Daems, T.; Waer, M.; Herdewijn, P.; Louat, T.; Steinmetz, M.O.; De Jonghe, S.; Sprangers, B.; Daelemans, D. 3-nitropyridine analogues as novel microtubule-targeting agents. *PLoS One* **2024**, *19*, e0307153, doi:10.1371/journal.pone.0307153.
52. Jiang, F.; Yu, M.; Wang, Y. Design, synthesis and biological evaluation of novel diaryl-substituted fused nitrogen heterocycles as tubulin polymerization inhibitors to overcome multidrug resistance in vitro and in vivo. *Eur J Med Chem* **2025**, *283*, 117130, doi:10.1016/j.ejmech.2024.117130.
53. Marzullo, P.; Boiarska, Z.; Perez-Pena, H.; Abel, A.C.; Alvarez-Bernad, B.; Lucena-Agell, D.; Vasile, F.; Sironi, M.; Altmann, K.H.; Prota, A.E.; Diaz, J.F.; Pieraccini, S.; Passarella, D. Maytansinol Derivatives: Side Reactions as a Chance for New Tubulin Binders. *Chemistry* **2022**, *28*, e202103520, doi:10.1002/chem.202103520.
54. Yang, M.H.; Mao, J.; Zhu, J.H.; Zhang, H.; Ding, L. Wangzaozin A, a potent novel microtubule stabilizer, targets both the taxane and laulimalide sites on beta-tubulin through molecular dynamics simulations. *Life Sci* **2022**, *301*, 120583, doi:10.1016/j.lfs.2022.120583.
